# Comparative Genomics of the *Rhodococcus* Genus Shows Wide Distribution of Biodegradation Traits

**DOI:** 10.3390/microorganisms8050774

**Published:** 2020-05-21

**Authors:** Daniel Garrido-Sanz, Miguel Redondo-Nieto, Marta Martín, Rafael Rivilla

**Affiliations:** Departamento de Biología, Facultad de Ciencias, Universidad Autónoma de Madrid, Darwin 2, 28049 Madrid, Spain; daniel.garrido@uam.es (D.G.-S.); miguel.redondo@uam.es (M.R.-N.); m.martin@uam.es (M.M.)

**Keywords:** *Rhodococcus*, comparative genomics, phylogenomics, biodegradation

## Abstract

The genus *Rhodococcus* exhibits great potential for bioremediation applications due to its huge metabolic diversity, including biotransformation of aromatic and aliphatic compounds. Comparative genomic studies of this genus are limited to a small number of genomes, while the high number of sequenced strains to date could provide more information about the *Rhodococcus* diversity. Phylogenomic analysis of 327 *Rhodococcus* genomes and clustering of intergenomic distances identified 42 phylogenomic groups and 83 species-level clusters. Rarefaction models show that these numbers are likely to increase as new *Rhodococcus* strains are sequenced. The *Rhodococcus* genus possesses a small “hard” core genome consisting of 381 orthologous groups (OGs), while a “soft” core genome of 1253 OGs is reached with 99.16% of the genomes. Models of sequentially randomly added genomes show that a small number of genomes are enough to explain most of the shared diversity of the *Rhodococcus* strains, while the “open” pangenome and strain-specific genome evidence that the diversity of the genus will increase, as new genomes still add more OGs to the whole genomic set. Most rhodococci possess genes involved in the degradation of aliphatic and aromatic compounds, while short-chain alkane degradation is restricted to a certain number of groups, among which a specific particulate methane monooxygenase (pMMO) is only found in *Rhodococcus* sp. WAY2. The analysis of Rieske 2Fe-2S dioxygenases among rhodococci genomes revealed that most of these enzymes remain uncharacterized.

## 1. Introduction

*Rhodococcus* is a gram-positive genus within the *Actinobacteria* class that is ubiquitously distributed in the environment. Strains from this genus have been isolated from a variety of habitats, including soils, oceans and fresh waters [1,2,3], as well as from the guts of insects or living in association with sea sponges [4,5]. Some species are known pathogens, including *R. hoagii* (formerly *R. equi*), which causes zoonotic infections in grazing animals [6,7], and *R. fascians*, the causing agent of leafy gall disease in plants [8,9]. In addition, multiple *Rhodococcus* species are known to degrade diverse organic compounds, including polychlorinated biphenyls (PCBs), polycyclic aromatic hydrocarbons (PAHs) and aliphatic hydrocarbons [10,11,12], making this genus a very promising tool for bioremediation purposes. The diverse number of niches that rhodococci are able to inhabit and their extensive catabolic potential are thought to be a consequence of their large genomes and the presence of multiple extrachromosomal elements that add new functional traits to the general content [13].

The taxonomy of the *Rhodococcus* genus is constantly changing, due to the frequent description of novel species [14,15,16], which adds more complexity to the frequent reassignments and merge of species [17]. Examples of the inconsistency in the classification can be found in the report of an illegitimate genus name of *Rhodococcus* Zopf 1981 which postdates the homonym algal genus *Rhodococcus* Hansgirg 1884 [18], and the proposed reclassification of *Rhodococcus equi* to the genus *Prescottia* [19]. However, the formal reclassification of *R. equi* into the species *Rhodococcus hoagii* [17] has further complicated this question, which awaits formal consideration [20]. Until these issues are resolved, *Rhodococcus hoagii* is still valid [21] and also the genus *Rhodococcus* Zopf 1981, which currently includes 66 validly named species, according to the List of Prokaryotic names with Standing in Nomenclature [22] (accessed in July 2019).

Phylogenies of the *Rhodococcus* genus based on multilocus sequence analysis (MLSA) using the housekeeping genes 16S rRNA, *secY*, *rpoC*, and *rpsA* [23] or several universal protein sequences [20] have been used to address the phyletic relationship within strains from this genus and to identify a varying number of groups of species [20,24], providing more reliability than phylogenies based on the 16S rRNA gene [25,26]. However, the number of sequenced rhodococci allows now the use of whole-genome comparisons for a better understanding of their relatedness and divergence. In this sense, Average Nucleotide Identity (ANI) [27] has been used to identify seven clades within 59 *Rhodococcus* isolates [25], although in other proteobacterial genera, including *Pseudomonas* and *Bradyrhizobium*, the genome-to-genome blast distance phylogeny (GBDP) algorithm [28] has proven to be more reliable than ANI for establishing species and phylogenomic groups boundaries [29,30]. Comparative genomics have also been performed to assess the functional diversity of several rhodococcal groups [23,24]. However, these analyses are scarce and limited to a few genome comparisons, which do not represent the entire diversity of the genus. Therefore, a global comparison of *Rhodococcus* genomes is needed to better understand the differences in their lifestyles and catabolic potential and to further acknowledge their diversity.

Among the different members of the *Rhodococcus* genus, we previously isolated the novel PCB degrader *Rhodococcus* sp. WAY2 from a biphenyl-degrading bacterial consortium [31]. Further analysis of its complete genome sequence revealed several genetic clusters and genes putatively involved in the biodegradation of various aromatic compounds and different chain-length alkanes [32]. Although most of these clusters have also been reported in other rhodococci [10,11,33], the distribution of these biodegradative traits among the *Rhodococcus* genus remains unexplored.

In this work, we report a global comparative genomic study of the *Rhodococcus* genus, using more than 300 sequenced strains. By means of phylogenomics, digital DNA–DNA hybridization (dDDH) and the determination of clusters of orthologous groups (OGs), we explore its diversity. Finally, we analyze the distribution of certain genes and gene clusters relevant for the biodegradation of aromatic and aliphatic compounds among *Rhodococcus* genomes to characterize their distribution among the genus.

## 2. Materials and Methods

### 2.1. Datasets

All sequenced *Rhodococcus* genomes, proteomes, and annotations were downloaded from the RefSeq (GeneBank when RefSeq not available) NCBI ftp server [34] in June 2019. Duplicated type strain genomes from different culture collections were removed based on the number of contigs, removing those with a higher number, likely underrepresenting the strain genome, resulting in a total of 327 genomes listed in Supplementary Table S1.

### 2.2. Phylogenomic Analysis

The 327 *Rhodococcus* genomes were compared using the Genome-to-genome Blast Distance Phylogeny (GBDP) algorithm [28] via the Genome-to-genome Distance Calculator (GGDC) web service [35]. The resulting sets of intergenomic distances (Supplementary Table S2) were converted into a matrix and imported into MEGA X software [36] to build a Neighbor–Joining (NJ) phylogenomic tree. *Nocardia brasiliensis* ATCC 700358 was used as outgroup. In addition, GBDP was also used to calculate the digital DNA–DNA hybridization (dDDH) values among all genome pairwise comparisons.

### 2.3. Clustering of Rhodococcus Genomes

Clustering of GBDP intergenomic distances from the *Rhodococcus* genus at species level (70% dDDH) and into phylogenomic groups was examined using the OPTSIL clustering software (version 1.5, Available online: http://www.goeker.org/mg/clustering/) [37]. An average-linkage clustering (i.e., *F* = 0.5) was chosen, as previously proposed [29,38] and clustering threshold (*T*) values from 0 to 0.2, using a step size of 0.0005 were evaluated. The best *T* for both species and phylogenomic groups were selected based on reference partitions that yielded the highest Modified Rand Index (MRI) score, used to measure the stability of similarity of partitions.

Interpolation and extrapolation analyses of the species and phylogenomic groups clusters were inferred using the iNEXT R package [39], with a bootstrap of 1000 replicates and a confidence interval of 95%.

### 2.4. Orthologous Groups Identification and Genome Fractions

Given the large number of genomes used in the study, for the identification of orthologous groups, genomes with more than 75 scaffolds (90 genomes) were removed to avoid misrepresentation of genomic fractions. Proteomes of the 237 resulting *Rhodococcus* genomes were compared using the OrthoFinder software (version 2.3.3, Available online: https://github.com/davidemms/OrthoFinder) [40], using diamond [41] searchers and the MCL graph clustering algorithm [42]. Resulting orthologous clusters were queried with an in-house designed R script to obtain the core, pangenome, and group-specific genome fractions over 300 randomly sampled genomes (i.e., 300 indices of the 237 genomes randomly selected, were constructed and queried independently to obtain the number of orthologous groups of each genome fraction). The mean, Q1, and Q3 statistics of the 300 curves for each genome fraction were calculated and then represented using the ggplot2 R package [43]. The orthologous groups identified and the R script used to calculate the genome fractions have been included in the Supplementary File S1 and Supplementary File S2, respectively. Hierarchical clustering of selected orthologous groups was performed using the pheatmap R package [44].

### 2.5. Phylogeny of Single-Copy Genes

Orthologous sequences of 212 single copy genes present in all the genomes were used to construct a phylogenetic tree. Amino acid sequences of the 212 single copy genes were aligned using the Clustal Omega software [45] and then concatenated. The resulting alignment of concatenated sequences was examined to remove poorly aligned columns and highly divergent regions with the gblocks v0.91 software [46], using a minimum block length of two amino acids and allowing gap positions in all sequences. The resulting matrix was imported into the Pthreads-parallelized RAxML v8.2.12 [47] to build a maximum-likelihood (ML) phylogenetic tree, using the LG model of amino acid evolution [48] combined with gamma-distributed substitution rates and empirical frequencies of amino acids. Fast bootstrapping was applied, followed by the search for the best-scoring tree [49] and the autoMRE criterium [50] were applied. Tree inference was calculated using the CIPRES Science Getaway [51]. Results were imported into MEGA X software to draw the tree.

### 2.6. Diversity of Rieske 2Fe-2S Dioxygenases

The orthologous group containing Rieske 2Fe-2S dioxygenase homologous sequences previously identified was used to construct a maximum-likelihood phylogenetic tree, using the same methods and parameters specified above. Identical sequences were removed, and highly divergent regions were conserved to avoid the removal of divergent sequences given the diversity of the sequences analyzed.

## 3. Results and Discussion

### 3.1. Phylogenomic Analysis and Clustering of the Rhodococcus Genus

**Figure 1:**
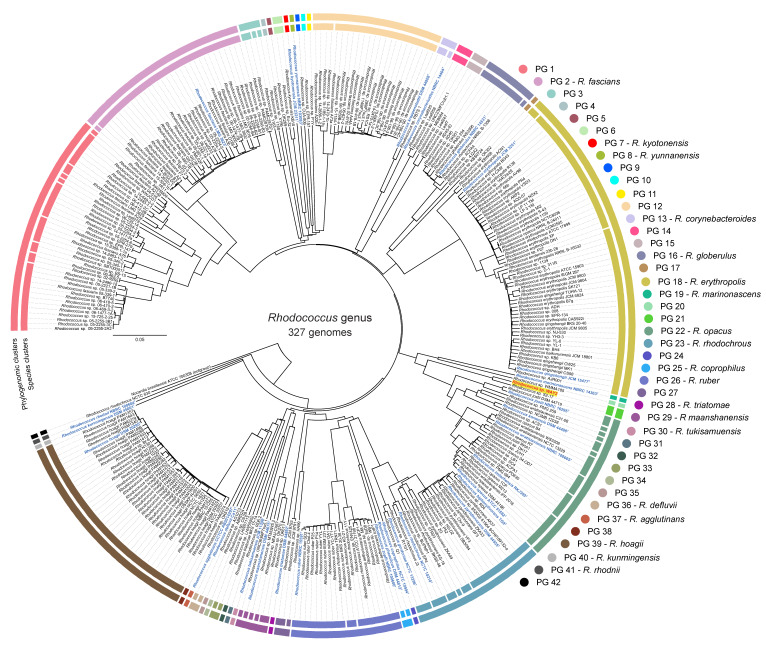
Genome-to-genome blast distance phylogeny (GBDP)-based phylogeny of 327 Rhodococcus genomes. The neighbor-joining tree was built using the GBDP intergenomic distances. *Nocardia brasiliensis* ATCC 700358 was used as outgroup. Clusters at the species level (inner circle) or phylogenomic groups (PGs, outer circle) are defined by OPTSIL clustering of intergenomic distances. Colors according to PG. Blue, bold and ^T^ indicate type strain. *Rhodococcus* sp. WAY2 is highlighted in yellow and red typing.

**Figure 2:**
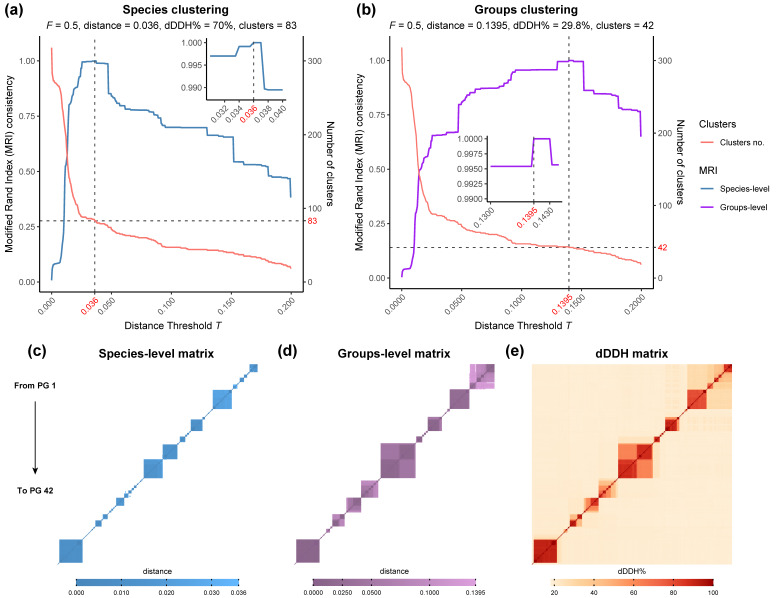
Clustering analysis of 327 *Rhodococcus* genomes using a range of distance thresholds *T*. Total cluster consistency (i.e., MRI = 1) was achieved using an average linkage (i.e., *F* = 0.5) at both species-level (**a**) and groups-level clusters (**b**) compared to the reference partition. Clustering was performed with the OPTSIL software v1.5 [37]. Distance matrices (**c**,**d**) and digital DNA–DNA hybridization (dDDH) matrix (**e**) show these clusters from PG 1 (upper-right) to PG 42 (lower-left).

The phylogenomic GBDP-based analysis of 327 *Rhodococcus* genomes and further clustering of the intergenomic distances (Supplementary Table S1) revealed the presence of 42 phylogenomic groups (PGs) and 83 species-level clusters (Figure 1 and Figure 2). The 42 PGs are in total agreement with the reference partition according to the Modified Rand Index (i.e., MRI = 1) using a distance threshold *T* between 0.1395 and 0.143, which correspond to a 29.8% and 30.5% dDDH, respectively (Figure 2). This result is similar to the threshold identified for phylogroups clustering in the genera *Pseudomonas* and *Bradyrhizobium* (Garrido-Sanz et al., 2016; Garrido-Sanz et al., 2019). These 42 PGs contain 22 single-genome clusters, some of which are composed of a type strain alone, and 20 others with more than one genome. Only 18 PGs contain type sequenced strain genomes and, according to the oldest species description, these are named *R. fascians*, (PG 2), *R. kyotonensis* (PG 7), *R. yunnanensis* (PG 8), *R. corynebacterioides* (PG 13), *R. globerulus* (PG 16), *R. erythropolis* (PG 18), *R. marinonascens* (PG 19), *R. opacus* (PG 22), *R. rhodochrous* (PG 23), *R. coprophilus* (PG 25), *R. ruber* (PG 26), *R. triatomae* (PG 28), *R. maashanensis* (PG 29), *R. tukisamuensis* (PG 30), *R. defluvii* (PG 36), *R. agglutinans* (PG 37), *R. hoagii* (PG 39), *R. kunmingensis* (PG 40), and *R. rhodnii* (PG 41, Figure 1). The genome of *Rhodococcus* sp. WAY2 [32] is clustered with *Rhodococcus* sp. S2-17 and corresponds to the PG 21. Some of the PGs identified in this work are in agreement with a previous study conducted by Creason et al., 2014, which identified seven main clades within the *Rhodococcus* genus using 59 genomes based on whole-genome comparisons [25]. Clade I corresponds to PG 1 (sub-clades ii, iii and iv) and PG 2-*R. fascians* (sub-clade i), and clade II corresponds to PG 12. These two clades were phylogenetically close, as is the case of the PG 1 to PG 12 in our analyses, which share an ancestral node. Clades III, IV, V, VI, and VII identified by Creason et al., 2014 correspond to PG 18-*R. erythropolis* (clade III), PG 22-*R. opacus* (clade IV), PGs 39, 40 and 41 (*R. hoagii*, *R. kunmingensis* and *R. rhodnii*, all included in clade V), PG 26-*R. ruber* (clade VI), and PG 23-*R. rhodochrous* (clade VII), respectively. The remaining PGs identified in our analysis are probably missing from the previous study due to their smaller dataset. However, the fact that both analyses found the same phylogenomic groups supports their status.

On the other hand, we identified 83 species-level clusters within the 327 *Rhodococcus* genomes. These clusters were established with the conventional threshold of 70% dDDH, which corresponds to a distance of 0.036 between genomes. The clustering result is in total agreement with the reference partition (i.e., MRI = 1, Figure 2). Thirty of these clusters contain sequenced type strains genomes, while the remaining 53, either correspond to previously not sequenced type strains or are novel species, which should be properly validated in accordance with standards in nomenclature. Surprisingly, several genomes of type strain species clustered together, achieving dDDH% values higher than 70% (Supplementary Table S2). These include *R. imtechensis* RKJ300^T^ and *R. opacus* ATCCC 51882^T^ (80.2% dDDH, 77.3–82.9% confidence interval and 90.77% probability of same species) and *R. biphenylivorans* TG9^T^ and *R. pyridinivorans* DSM 44555^T^ (88.3% dDDH, 85.9–90.4% confidence interval and 95.2% probability of belonging to the same species), whose species status should be properly revised. In addition, *Rhodococcus* sp. WAY2 achieved a 70.2% dDDH with *Rhodococcus* sp. S2-17, with a 67.2%–73% confidence interval and a 78.63% probability of same species.

**Figure 3:**
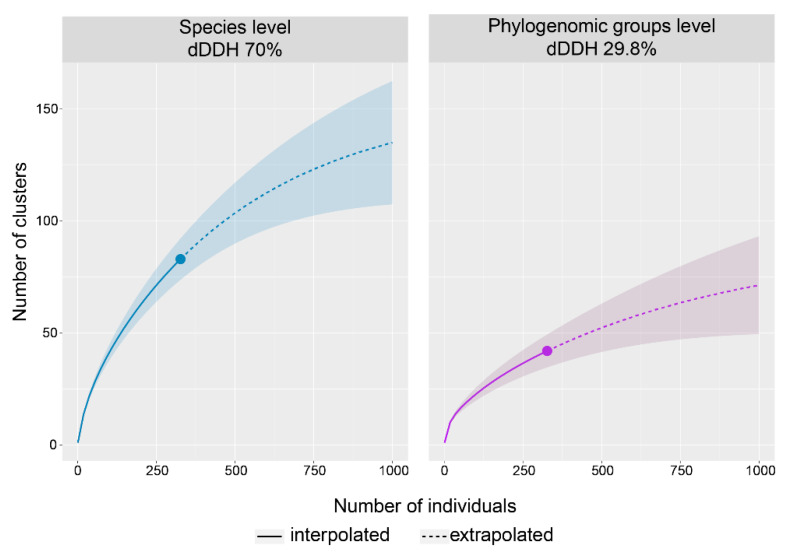
Interpolation/extrapolation rarefaction analysis of the clusters at species and phylogenomic groups levels (left and right respectively), using 1000 replicates and a 95% confidence interval.

In order to investigate whether the diversity of PGs and species found within the *Rhodococcus* sequenced genomes had achieved its maximum representation, we conducted rarefaction analyses. The results are shown in Figure 3. In both cases, curves are far from reaching an asymptote with 327 genomes sampled, and extrapolation analysis up to 1000 genomes still shows an increment in the number of clusters, which will probably grow to the hundreds in the case of species and above 50 in the case of PGs (Figure 3). This is evidence that the diversity exhibited by the *Rhodococcus* genus will increase as long as new genomes are sequenced and is in agreement with the fact that most of the PGs are composed of only one genome.

### 3.2. Phylogeny Based on Single-Copy Proteins

**Figure 4:**
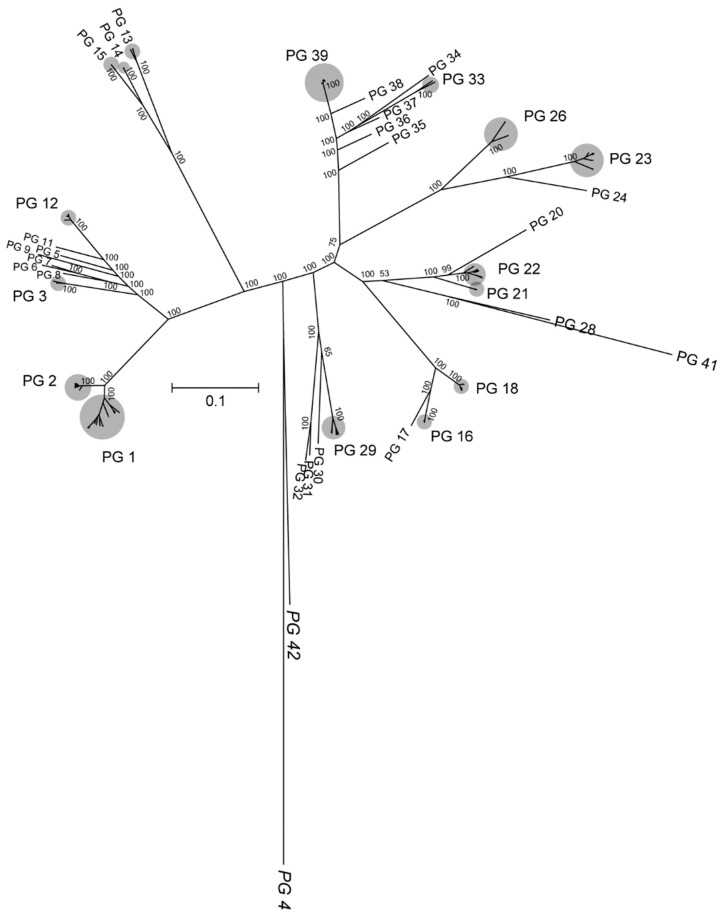
Maximum-likelihood phylogenetic tree of the *Rhodococcus* genus based on 212 single-copy amino acid sequences. PGs according to those identified in this study. Grey dots indicate PGs composed of multiple genomes. Bootstrap support is indicated above/below branches, not shown inside PGs.

The comparison of 237 strains proteomes resulted in a total of 17,258 orthologous groups (OGs). Among these OGs, 212 appeared in all the genomes as single-copy amino acid sequences. These OGs were used to construct a ML phylogenetic tree shown in Figure 4, whose clustering pattern is consistent with a previous phylogenetic analysis also based on amino acid sequences [20]. The same PGs found in the GBDP-based phylogenomic analysis (Figure 1) are also identified with total bootstrap support using amino acid sequences, which validates the genome clustering reported here. Nonetheless, PGs 4, 28, 41, and 42, all composed of single strains, are distant and separated from their closest PGs compared with the GBDP-based tree, probably due to different evolutionary pressure on the core fraction versus the whole genome content. In the case of PGs 41 and 42, composed of *R. rhodnii* NBRC 100604^T^ and *R. rhodochrous* NCTC 630, respectively, the high distance in the single-copy amino acid tree is also observed at the genomic level, being the earliest-diverging groups within the *Rhodococcus* genus (Figure 1). In addition, PG 41 and PG 28 (*R. rhodnii* NBRC 100604^T^ and *R. triatomae* DSM 44892^T^, respectively) are clustered together in the amino acid-based phylogeny, which agrees with a previous report [20]. In the specific case of PG 4 (composed of *Rhodococcus* sp. X156 genome), the unusually high GC% content (72.2) of this genome could result in a biased codon usage [52], which might explain the differences between the GBDP-based tree and its high divergence in the amino acid-based phylogeny.

Aside from these differences, both the GBDP and the amino acid-based analyses show a robust PG identity, maintain the same strain composition, and a similar phyletic pattern.

### 3.3. Genome Fractions of the Rhodococcus Genus

**Figure 5:**
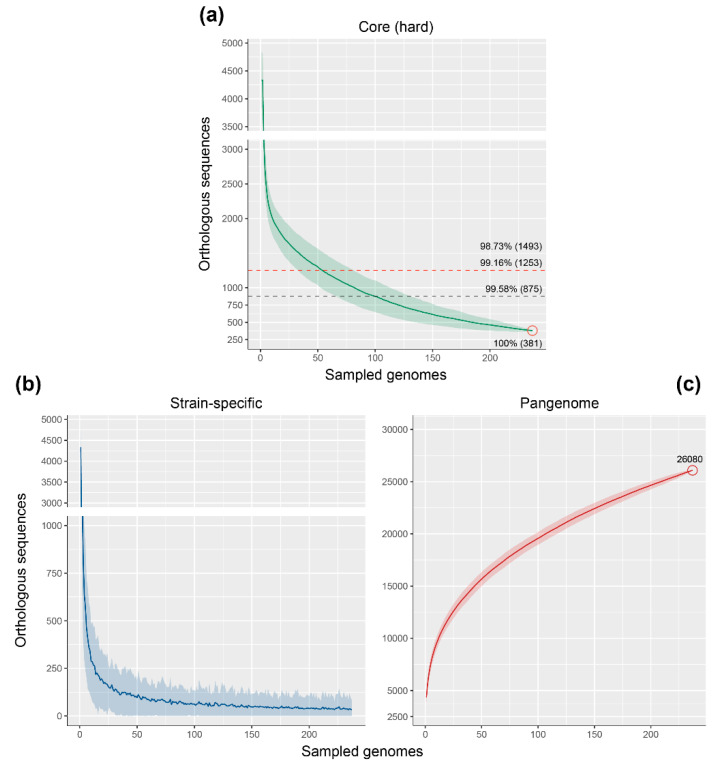
Genome fractions of the *Rhodococcus* genus. Core genome (**a**), strain-specific (**b**), and pangenome (**c**) analysis representing mean values (line) and Q1 and Q3 quantiles (shadow) over 300 replicates of 237 randomly sampled genomes. In the case of core genome, values at a different percentage of genomes sampled is indicated with dashed lines. Red circles in (a) and (c) indicate the maximum number of OGs achieved (below/above the red circle).

The orthologous groups identified by the comparative analysis were used to identify the core genome, the pangenome and the strain-specific genome fractions. The core genome of the *Rhodococcus* genus, which consists of those OGs which are represented in all genomes (“hard” core), is composed of only 381 OGs (Figure 5a). However, given the number of genomes included in the study, a “soft core” where a high percentage of genomes are represented, rather than the 100%, is probably more accurate. Considering a presence in at least 99.16% of the genomes, we obtain a soft core of 1253 OGs that shifts to 1493 OGs when fixing the threshold to 98.73% of genomes (Figure 5a).

Although there is no previous attempt to analyze the core genome of the *Rhodococcus* genus, but rather of certain groups [23,24], the number of “soft core” OGs is similar to that of other *Actinobacteria* genera. For example, analysis of 21 *Mycobacterium* genomes resulted in a core genome composed of ca. 1250 OGs [53], while 17 *Streptomyces* species (different bacterial order than *Rhodococcus* and *Mycobacterium*) present a core of 2018 OGs [54]. Core-genome size depending on the number of genomes sampled, as represented in Figure 5a, shows a rapid decrease in the number of OGs within the first randomly sampled genomes, and an asymptote is almost reached when considering the total 237 genomes used in the study.

The strain-specific genome fraction, represented as a function of the number of new OGs over sequentially added genomes (Figure 5b), also shows a rapid reduction within the first 50 sampled genomes, and then slowly decreases to reach an average of 33 OGs within 237 genomes. This implies that within 50 genomes, most of the *Rhodococcus* shared genetic content is achieved and more genomes would only add specific sequences, which is congruent with the 42 PGs identified in this study. However, the fact that on average each *Rhodococcus* adds 33 specific OGs and the high standard deviation observed in the strain-specific curve (Figure 5b) indicate that more genomes will keep increasing the overall genetic diversity of *Rhodococcus*. This is further evidenced in the pangenome curve, which reaches 26,080 OGs within the 237 sampled genomes (Figure 5c) and keeps a positive slope, being an “open” pangenome. The pangenome size of the *Rhodococcus* genus is similar to that reported in *Mycobacterium* and *Streptomyces*, composed of ca. 20,000 and 34,592 OGs, respectively [53,54].

### 3.4. Distribution of PAHs and Alkane Degradation Genes

**Figure 6:**
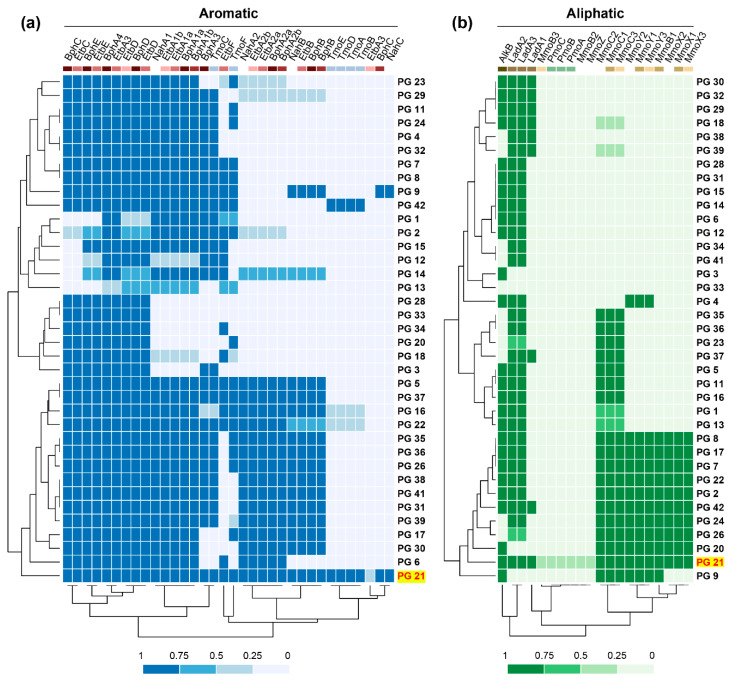
Distribution of orthologous groups (OGs) involved in aromatic (**a**) and aliphatic (**b**) compound degradation in *Rhodococcus* PGs. Color scale according to the fraction of genomes within each PG with the OG present. Colored boxes below enzyme names according to their cluster pattern in *Rhodococcus* sp. WAY2 (PG 21, highlighted in yellow and red typing).

*Rhodococcus* strains have the ability of degrading multiple organic compounds, including PAHs, dioxin and dioxin-like compounds, and different chain-length *n*-alkanes [10,11,12]. Degradation of aromatic compounds is commonly carried out by Rieske 2Fe-2S dioxygenase systems, including those involved in biphenyl/PCBs, ethylbenzene, and naphthalene degradation (*bph*, *etb* and *nah* gene clusters), which present a wide range of substrate specificity and have been reported in multiple *Rhodococcus* strains [11,33,55,56]. *Rhodococcus* genomes can simultaneously possess several of these systems [13,32]. Among them, *Rhodococcus* sp. WAY2 contains 5 different clusters putatively involved in the degradation of many aromatic compounds and a *tmo* gene cluster putatively involved in the conversion of toluene into *p*-cresol [32,57]. The OGs, which include the genes of these clusters in WAY2, were searched to address their distribution within the *Rhodococcus* genus and are shown in Figure 6a. Alpha subunits of these Rieske 2Fe-2S dioxygenases (BphA1a, BphA1b, EtbA1a, EtbA1b, and NahA1) are widely distributed within the genus PGs. However, they are missing from PGs 3, 20, 28, 33, and 34, and partially present in PGs 12, 13, and 18. Interestingly, the beta subunits of these dioxygenases (BphA2a, BphA2b, EtbA2a, EtbA2b, and NahA2a) have a more discrete distribution, being only present in 15 PGs (5, 6, 16, 17, 21, 22, 26, 30, 31, 35, 36, 37, 38, 39, and 41) and partially present in another 4 PGs (2, 14, 23 and 29), which also harbor the alpha subunits (Figure 6a). These PGs contain known degraders of aromatic compounds, including *R. jostii* RHA1 (PG 22) [13] and *Rhodococcus* sp. WAY2 (PG 21) [32]. This finding suggests that the degradation of aromatic compounds might be restricted to these PGs, at least of those compounds whose biodegradation is initiated by Rieske ring-hydroxylating dioxygenases of the orthologous group analyzed. On the other hand, the *tmo* gene cluster involved in the conversion of toluene to *p*-cresol [57] has a more limited distribution, being only present in PGs 42 and 21 and partially present in PGs 16 and 22 (Figure 6a), suggesting a specialized and distinctive metabolism of aromatic compounds in strains from these groups.

Aliphatic compounds, on the other hand, can be degraded by several different pathways [58]. The first step is a monooxygenation catalyzed by soluble or particulate methane monooxygenases (sMMO or pMMO, respectively) for short chain *n*-alkanes [59,60], or alkane monooxygenases (AlkB) and long-chain alkane monooxygenases (LadA) for middle and long-chain *n*-alkanes, respectively [58,61,62,63]. The distribution of orthologous sequences of these genes and gene clusters within *Rhodococcus* PGs shows an interesting pattern (Figure 6b). AlkB and LadA are found in most of the PGs (except PG 33, which does not harbor any of these genes), which suggests that almost all *Rhodococcus* strains could putatively degrade middle to long-chain *n*-alkanes. Conversely, sMMO subunits are present in a more limited number of groups (PGs 8, 2, 7, 9, 17, 20, 21, 22, 24, 26, and 42). Interestingly, mmoC, which encodes the iron–sulfur component of sMMO [64], is found in other groups that do not contain the remaining sMMO subunits (Figure 6b). This could be explained by similar homology to other iron–sulfur electron transfer systems. Surprisingly, the pMMO system reported in *Rhodococcus* sp. WAY2 [32] is not found in any other PG or genome within the *Rhodococcus* genus, being a unique and distinctive feature of WAY2 (Figure 6b). It has been reported that pMMO has a narrow substrate specificity, oxidizing *n*-alkanes up to C_5_, preferentially at the C_2_ position [65], and it has been found in several putative aerobic methanotrophic bacteria [66]. The absence of this cluster in other rhodococci could imply a horizontal transfer event and a novel catabolic acquisition that distinguish this strain from any other *Rhodococcus*, although further analyses are required to prove this hypothesis and test its functionality in *Rhodococcus* sp. WAY2.

Nonetheless, although, in this study, only the distribution of the main traits reported in *Rhodococcus* sp. WAY2 have been explored, other traits not found in WAY2 could also show a distinctive pattern among the rest of PGs in the genus, which require further analysis.

### 3.5. Diversity of Rieske 2Fe-2S Dioxygenases among Rhodococcus Genomes

**Figure 7:**
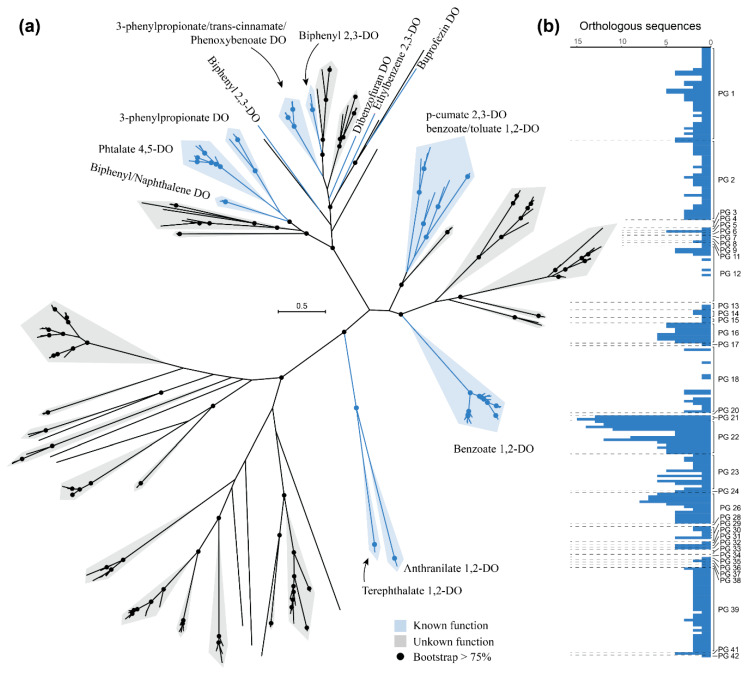
Rieske 2Fe-2S dioxygenase phylogenetic tree (**a**) and abundance of orthologous sequences (**b**) among *Rhodococcus* PGs. The unrooted maximum-likelihood tree was constructed with 339 unique sequences found in the OG with Rieske 2Fe-2S dioxygenases. Sequences of dioxygenases (DO) with known function/substrate are highlighted in blue, and those without known function/substrate are highlighted in grey. Dots indicate bootstrap support higher than 75%. The number of orthologous Rieske 2Fe-2S dioxygenases found in each analyzed *Rhodococcus* genome are represented in the barplot.

The diversity of Rieske 2Fe-2S dioxygenases has been previously analyzed, either in well-known and characterized sequences from different taxa [67,68] or in environmental samples [69,70]. We used the orthologous group containing these dioxygenases in the *Rhodococcus* genus to construct a phylogenetic tree, to assess their diversity within the genus. The orthologous group of these dioxygenases contains 567 sequences, of which 339 are not identical and were used to construct the phylogeny (Figure 7a). These sequences include biphenyl 2,3-dioxygenases, naphthalene 1,2-dioxygenases, ethylbenzene 2,3-dioxygenases, phthalate 4,5-dioxygenases, 3-phenylpropionate dioxygenases, benzoate 1,2-dioxygenases, and other dioxygenases with known substrates (Figure 7a). Surprisingly most of the sequences constitute large and very diverse groups without known function/substrate annotated to date. All the sequences involved in the degradation of peripheral substrates (biphenyl, naphthalene, and ethylbenzene, among others) clustered together, along with certain groups of proteins with unknown substrate. Other groups of sequences involved in central aromatic metabolism (benzoate) or central nodes in aromatic degradation pathways (*p*-cumate, anthranilate, and terephthalate) also form distinct clusters. From the total of 339 unique sequences analyzed, the substrates of more than 200 remain unknown.

On the other hand, the number of orthologs found in each of the genomes analyzed differs widely (Figure 7b). The PGs that harbor the highest number of Rieske 2Fe-2S dioxygenases are those of known degraders of aromatic compounds. For example, *R. jostii* RHA1 [13], *R. opacus* strains B4 [71], and R7 [72], all included in PG 22, contain 12, 11, and 9 Rieske 2Fe-2S dioxygenase orthologs, respectively. Similarly, *Rhodococcus* sp. WAY2 [32] and *Rhodococcus* sp. S2-17, forming PG 21, contain 13 and 15 of these dioxygenases, respectively (Figure 7b), S2-17 being the strain with the highest number of Rieske 2Fe-2S dioxygenases identified. Therefore, there are probably novel functions and substrates that remain undiscovered within the large number of uncharacterized dioxygenases present in *Rhodococcus* genomes, which is consistent with the diversity of novel and not functionally characterized dioxygenases usually found in environmental studies [69,70].

## 4. Conclusions

The diversity of the *Rhodococcus* genus is reflected in the 42 phylogenomic groups (PGs) and 83 species clusters that are identified within more than 300 sequenced genomes. The number of PGs and species are likely to increase with the sequencing of more strains. Comparative genomic analysis shows a high degree of genetic diversity reflected in a small core genome of 381 orthologous groups and a large open pangenome of 26,080 PGs. The distribution of biodegradative traits among *Rhodococcus* PGs shows that although many of the *Rhodococcus* strains could potentially catabolize aromatic and aliphatic compounds, short-chain n-alkanes biodegradation is limited to a certain number of groups, and specialized metabolism of these alkanes is present in *Rhodococcus* sp. WAY2. Finally, the high number and diversity of Rieske 2Fe-2S dioxygenases with unknown substrate among rhodococci genomes makes the discovery of novel aromatic compounds’ degradation a possibility that requires further exploration.

## Supplementary Materials

The following are available online at https://www.mdpi.com/2076-2607/8/5/774/s1, Table S1: List of *Rhodococcus* genomes used in this study, Table S2: GGDC intergenomic distances of the pairwise *Rhodococcus* genome comparisons, File S1: Orthologous groups identified among the *Rhodococcus* genomes, and File S2: R code used to obtain the genome fractions.

## References

[1] Helmke E., Weyland H. (1984). *Rhodococcus marinonascens* sp. nov., an actinomycete from the sea. Int. J. Syst. Evol. Microbiol..

[2] Margesin R., Labbe D., Schinner F., Greer C., Whyte L. (2003). Characterization of hydrocarbon-degrading microbial populations in contaminated and pristine alpine soils. Appl. Environ. Microbiol..

[3] Ryu H.-W., Joo Y.-H., An Y.-J., Cho K.-S. (2006). Isolation and characterization of psychrotrophic and halotolerant *Rhodococcus* sp. YHLT-2. J. Microbiol. Biotechnol..

[4] Adnani N., Braun D.R., McDonald B.R., Chevrette M.G., Currie C.R., Bugni T.S. (2016). Complete genome sequence of *Rhodococcus* sp. strain WMMA185, a marine sponge-associated bacterium. Genome Announc..

[5] Yassin A. (2005). *Rhodococcus triatomae* sp. nov., isolated from a blood-sucking bug. Int. J. Syst. Evol. Microbiol..

[6] Giguère S., Cohen N., Keith Chaffin M., Hines S., Hondalus M., Prescott J., Slovis N. (2011). *Rhodococcus equi*: Clinical Manifestations, Virulence, and Immunity. J. Vet. Intern. Med..

[7] Prescott J.F. (1991). *Rhodococcus equi*: An animal and human pathogen. Clin. Microbiol. Rev..

[8] Cornelis K., Ritsema T., Nijsse J., Holsters M., Goethals K., Jaziri M. (2001). The plant pathogen *Rhodococcus fascians* colonizes the exterior and interior of the aerial parts of plants. Mol. Plant-Microbe Interact..

[9] Goethals K., Vereecke D., Jaziri M., Van Montagu M., Holsters M. (2001). Leafy gall formation by *Rhodococcus fascians*. Annu. Rev. Phytopathol..

[10] De Carvalho C.C., Parreño-Marchante B., Neumann G., Da Fonseca M.M.R., Heipieper H.J. (2005). Adaptation of *Rhodococcus erythropolis* DCL14 to growth on *n*-alkanes, alcohols and terpenes. Appl. Microbiol. Biotechnol..

[11] Iwasaki T., Takeda H., Miyauchi K., Yamada T., Masai E., Fukuda M. (2007). Characterization of two biphenyl dioxygenases for biphenyl/PCB degradation in a PCB degrader, *Rhodococcus* sp. strain RHA1. Biosci. Biotechnol. Biochem..

[12] Song X., Xu Y., Li G., Zhang Y., Huang T., Hu Z. (2011). Isolation, characterization of *Rhodococcus* sp. P14 capable of degrading high-molecular-weight polycyclic aromatic hydrocarbons and aliphatic hydrocarbons. Mar. Pollut. Bull..

[13] McLeod M.P., Warren R.L., Hsiao W.W., Araki N., Myhre M., Fernandes C., Miyazawa D., Wong W., Lillquist A.L., Wang D. (2006). The complete genome of *Rhodococcus* sp. RHA1 provides insights into a catabolic powerhouse. Proc. Natl. Acad. Sci. USA.

[14] Lee S.D., Kim Y.-J., Kim I.S. (2019). *Rhodococcus subtropicus* sp. nov., a new actinobacterium isolated from a cave. Int. J. Syst. Evol. Microbiol..

[15] Silva L.J., Souza D.T., Genuario D.B., Hoyos H.A.V., Santos S.N., Rosa L.H., Zucchi T.D., Melo I.S. (2018). *Rhodococcus psychrotolerans* sp. nov., isolated from rhizosphere of *Deschampsia antarctica*. Antonie Van Leeuwenhoek.

[16] Wang L., Zhang L., Zhang X., Zhang S., Yang L., Yuan H., Chen J., Liang C., Huang W., Liu J. (2019). *Rhodococcus daqingensis* sp. nov., isolated from petroleum-contaminated soil. Antonie van Leeuwenhoek.

[17] Kämpfer P., Dott W., Martin K., Glaeser S.P. (2014). *Rhodococcus defluvii* sp. nov., isolated from wastewater of a bioreactor and formal proposal to reclassify [*Corynebacterium hoagii*] and *Rhodococcus equi* as *Rhodococcus hoagii* comb. nov. Int. J. Syst. Evol. Microbiol..

[18] Tindall B. (2014). A note on the genus name *Rhodococcus* Zopf 1891 and its homonyms. Int. J. Syst. Evol. Microbiol..

[19] Jones A., Sutcliffe I., Goodfellow M. (2013). Proposal to replace the illegitimate genus name *Prescottia* Jones et al. 2013 with the genus name *Prescottella* gen. nov. and to replace the illegitimate combination *Prescottia equi* Jones et al. 2013 with *Prescottella equi* comb. nov. Antonie van Leeuwenhoek.

[20] Sangal V., Goodfellow M., Jones A.L., Seviour R.J., Sutcliffe I.C. (2019). Refined Systematics of the Genus Rhodococcus Based on Whole Genome Analyses. Biology of Rhodococcus.

[21] Tindall B. (2014). The correct name of the taxon that contains the type strain of *Rhodococcus equi*. Int. J. Syst. Evol. Microbiol..

[22] Parte A.C. (2014). LPSN—list of prokaryotic names with standing in nomenclature. Nucleic Acids Res..

[23] Orro A., Cappelletti M., D’Ursi P., Milanesi L., Di Canito A., Zampolli J., Collina E., Decorosi F., Viti C., Fedi S. (2015). Genome and phenotype microarray analyses of *Rhodococcus* sp. BCP1 and *Rhodococcus opacus* R7: Genetic determinants and metabolic abilities with environmental relevance. PLOS ONE.

[24] Anastasi E., MacArthur I., Scortti M., Alvarez S., Giguère S., Vázquez-Boland J.A. (2016). Pangenome and phylogenomic analysis of the pathogenic actinobacterium *Rhodococcus equi*. Genome Biol. Evol..

[25] Creason A.L., Davis E.W., Putnam M.L., Vandeputte O.M., Chang J.H. (2014). Use of whole genome sequences to develop a molecular phylogenetic framework for *Rhodococcus fascians* and the *Rhodococcus* genus. Front. Plant Sci..

[26] Gürtler V., Mayall B.C., Seviour R. (2004). Can whole genome analysis refine the taxonomy of the genus *Rhodococcus*?. FEMS Microbiol. Rev..

[27] Richter M., Rosselló-Móra R. (2009). Shifting the genomic gold standard for the prokaryotic species definition. Proc. Natl. Acad. Sci. USA.

[28] Meier-Kolthoff J.P., Auch A.F., Klenk H.-P., Göker M. (2013). Genome sequence-based species delimitation with confidence intervals and improved distance functions. BMC Bioinform..

[29] Garrido-Sanz D., Meier-Kolthoff J.P., Göker M., Martin M., Rivilla R., Redondo-Nieto M. (2016). Genomic and genetic diversity within the *Pseudomonas fluorescens* complex. PLOS ONE.

[30] Garrido-Sanz D., Redondo-Nieto M., Mongiardini E., Blanco-Romero E., Durán D., Quelas J.I., Martin M., Rivilla R., Lodeiro A.R., Althabegoiti M.J. (2019). Phylogenomic analyses of *Bradyrhizobium* reveal uneven distribution of the lateral and subpolar flagellar systems, which extends to *Rhizobiales*. Microorganisms.

[31] Garrido-Sanz D., Manzano J., Martín M., Redondo-Nieto M., Rivilla R. (2018). Metagenomic analysis of a biphenyl-degrading soil bacterial consortium reveals the metabolic roles of specific populations. Front. Microbiol..

[32] Garrido-Sanz D., Sansegundo-Lobato P., Redondo-Nieto M., Suman J., Cajthaml T., Blanco-Romero E., Martin M., Uhlik O., Rivilla R. (2020). Analysis of the biodegradative and adaptive potential of the novel polychlorinated biphenyl degrader *Rhodococcus* sp. WAY2 revealed by its complete genome sequence. Microb. Genom..

[33] Kimura N., Kitagawa W., Mori T., Nakashima N., Tamura T., Kamagata Y. (2006). Genetic and biochemical characterization of the dioxygenase involved in lateral dioxygenation of dibenzofuran from *Rhodococcus opacus* strain SAO101. Appl. Microbiol. Biotechnol..

[34] NCBI ftp Server. ftp://ftp.ncbi.nlm.nih.gov.

[35] Genome-to-genome Distance Calculator (GGDC) 2.1. http://ggdc.dsmz.de/ggdc.php.

[36] Kumar S., Stecher G., Li M., Knyaz C., Tamura K. (2018). MEGA X: Molecular evolutionary genetics analysis across computing platforms. Mol. Biol. Evol..

[37] Göker M., García-Blázquez G., Voglmayr H., Tellería M.T., Martín M.P. (2009). Molecular taxonomy of phytopathogenic fungi: A case study in *Peronospora*. PLoS ONE.

[38] Meier-Kolthoff J.P., Hahnke R.L., Petersen J., Scheuner C., Michael V., Fiebig A., Rohde C., Rohde M., Fartmann B., Goodwin L.A. (2014). Complete genome sequence of DSM 30083 T, the type strain (U5/41 T) of *Escherichia coli*, and a proposal for delineating subspecies in microbial taxonomy. Stand. Genom. Sci..

[39] Hsieh T.C., Ma K.H., Chao A. (2016). iNEXT: An R package for rarefaction and extrapolation of species diversity (Hill numbers). Methods Ecol. Evol..

[40] Emms D.M., Kelly S. (2015). OrthoFinder: Solving fundamental biases in whole genome comparisons dramatically improves orthogroup inference accuracy. Genome Biol..

[41] Buchfink B., Xie C., Huson D.H. (2015). Fast and sensitive protein alignment using DIAMOND. Nat. Methods.

[42] Enright A.J., Van Dongen S., Ouzounis C.A. (2002). An efficient algorithm for large-scale detection of protein families. Nucleic Acids Res..

[43] Wickham H. (2011). ggplot2. Wiley Interdisciplinary Reviews. Comput. Stat..

[44] Kolde R., Kolde M.R. (2015). Package ‘pheatmap’. R Package.

[45] Sievers F., Wilm A., Dineen D., Gibson T.J., Karplus K., Li W., Lopez R., McWilliam H., Remmert M., Söding J. (2011). Fast, scalable generation of high-quality protein multiple sequence alignments using Clustal Omega. Mol. Syst. Biol..

[46] Castresana J. (2000). Selection of conserved blocks from multiple alignments for their use in phylogenetic analysis. Mol. Biol. Evol..

[47] Stamatakis A. (2014). RAxML version 8: A tool for phylogenetic analysis and post-analysis of large phylogenies. Bioinformatics.

[48] Le S.Q., Gascuel O. (2008). An improved general amino acid replacement matrix. Mol. Biol. Evol..

[49] Stamatakis A., Hoover P., Rougemont J. (2008). A rapid bootstrap algorithm for the RAxML web servers. Syst. Biol..

[50] Pattengale N.D., Alipour M., Bininda-Emonds O.R., Moret B.M., Stamatakis A. (2010). How many bootstrap replicates are necessary?. J. Comput. Biol..

[51] Miller M.A., Pfeiffer W., Schwartz T. Creating the CIPRES Science Gateway for inference of large phylogenetic trees. Proceedings of the 2010 Gateway Computing Environments Workshop (GCE).

[52] Li J., Zhou J., Wu Y., Yang S., Tian D. (2015). GC-content of synonymous codons profoundly influences amino acid usage. G3: Genes, Genomes, Genet..

[53] Zakham F., Aouane O., Ussery D., Benjouad A., Ennaji M.M. (2012). Computational genomics-proteomics and Phylogeny analysis of twenty one mycobacterial genomes (Tuberculosis & non Tuberculosis strains). Microbial Inform. Exp..

[54] Kim J.-N., Kim Y., Jeong Y., Roe J.-H., Kim B.-G., Cho B.-K. (2015). Comparative genomics reveals the core and accessory genomes of *Streptomyces* species. J. Microbiol. Biotechnol..

[55] Patrauchan M.A., Florizone C., Eapen S., Gomez-Gil L., Sethuraman B., Fukuda M., Davies J., Mohn W.W., Eltis L.D. (2008). Roles of ring-hydroxylating dioxygenases in styrene and benzene catabolism in *Rhodococcus jostii* RHA1. J. Bacteriol..

[56] Resnick S., Lee K., Gibson D. (1996). Diverse reactions catalyzed by naphthalene dioxygenase from *Pseudomonas* sp strain NCIB 9816. J. Ind. Microbiol..

[57] Yen K.-M., Karl M.R., Blatt L.M., Simon M.J., Winter R.B., Fausset P.R., Lu H.S., Harcourt A.A., Chen K.K. (1991). Cloning and characterization of a *Pseudomonas mendocina* KR1 gene cluster encoding toluene-4-monooxygenase. J. Bacteriol..

[58] Ji Y., Mao G., Wang Y., Bartlam M. (2013). Structural insights into diversity and *n*-alkane biodegradation mechanisms of alkane hydroxylases. Front. Microbiol..

[59] Elliott S.J., Zhu M., Tso L., Nguyen H.-H.T., Yip J.H.-K., Chan S.I. (1997). Regio-and stereoselectivity of particulate methane monooxygenase from *Methylococcus capsulatus* (Bath). J. Am. Chem. Soc..

[60] Smith T., Dalton H. (2004). Biocatalysis by methane monooxygenase and its implications for the petroleum industry. Studies in Surface Science and Catalysis.

[61] Johnson E.L., Hyman M.R. (2006). Propane and *n*-butane oxidation by *Pseudomonas putida* GPo1. Appl. Environ. Microbiol..

[62] Li L., Liu X., Yang W., Xu F., Wang W., Feng L., Bartlam M., Wang L., Rao Z. (2008). Crystal structure of long-chain alkane monooxygenase (LadA) in complex with coenzyme FMN: Unveiling the long-chain alkane hydroxylase. J. Mol. Biol..

[63] van Beilen J.B., Wubbolts M.G., Witholt B. (1994). Genetics of alkane oxidation by *Pseudomonas oleovorans*. Biodegradation.

[64] Stainthorpe A., Lees V., Salmond G.P., Dalton H., Murrell J.C. (1990). The methane monooxygenase gene cluster of *Methylococcus capsulatus* (Bath). Gene.

[65] Chan S.I., Chen K.H.-C., Yu S.S.-F., Chen C.-L., Kuo S.S.-J. (2004). Toward delineating the structure and function of the particulate methane monooxygenase from methanotrophic bacteria. Biochemistry.

[66] Tavormina P.L., Ussler W., Joye S.B., Harrison B.K., Orphan V.J. (2010). Distributions of putative aerobic methanotrophs in diverse pelagic marine environments. ISME J..

[67] Meynet P., Head I.M., Werner D., Davenport R.J. (2015). Re-evaluation of dioxygenase gene phylogeny for the development and validation of a quantitative assay for environmental aromatic hydrocarbon degraders. FEMS Microbiol. Ecol..

[68] Seeger M., Pieper D., Timmis K.N. (2010). Genetics of biphenyl biodegradation and co-metabolism of PCBs. Handbook of Hydrocarbon and Lipid Microbiology.

[69] Iwai S., Chai B., Sul W.J., Cole J.R., Hashsham S.A., Tiedje J.M. (2010). Gene-targeted-metagenomics reveals extensive diversity of aromatic dioxygenase genes in the environment. ISME J..

[70] Iwai S., Johnson T.A., Chai B., Hashsham S.A., Tiedje J.M. (2011). Comparison of the specificities and efficacies of primers for aromatic dioxygenase gene analysis of environmental samples. Appl. Environ. Microbiol..

[71] Grund E., Denecke B., Eichenlaub R. (1992). Naphthalene degradation via salicylate and gentisate by *Rhodococcus* sp. strain B4. Appl. Environ. Microbiol..

[72] Di Gennaro P., Terreni P., Masi G., Botti S., De Ferra F., Bestetti G. (2010). Identification and characterization of genes involved in naphthalene degradation in *Rhodococcus opacus* R7. Appl. Microbiol. Biotechnol..

