# Supplementary material for: Comparative Genomics of the Rhodococcus Genus Shows Wide Distribution of Biodegradation Traits
Source: Microorganisms. 2020 May 21;8(5):774. doi: 10.3390/microorganisms8050774 (PMC7285261; doi:10.3390/microorganisms8050774)
Supplement: Supplementary file 1 [file microorganisms-08-00774-s001.zip › Supplementary_File_S2.pdf]

Supplementary File S2. R code used to obtain the genome fractions.

This R code deals with how to prepare the data obtained by OrthoFinder software in order to obtain and analyse the genome fractions: core genome, strain-specific genome and pangenome and how to plot them. We'll use "dplyr" and "tidiverse" packages in order to avoid using SQL.

So first we will install and/or load the required packages.

```
library("dplyr")
```

1. Load Data

We only need one of the files produced by OrthoFinder analysis: "Orthogroups.GeneCount.tsv" This file contains a column for each strain and a row per orthogroup. The numbers wihin the cells contain the number of genes each strain has in that specific orthogroup. Before loading, modify this file according to the following:

1. Change all values > 0 by 1 (for the genome fractions calculations we don't mind paralogs)

2. Include singletons! which are stored in the Orthogroups.txt file. Minor processing required.

3. Remove the additional "TOTAL" column at the end of the file.

4. Save this file as Orthologs_and_singletons_01.txt (Supplementary_File_S1.txt in this case).

Let's load the data and take a look.

```
orthogroups <- read.csv("Supplementary_File_S1.txt", header=T, sep="\t")
head(orthogroups)
#To remove "X" from colnames
colnames(orthogroups) <- gsub('X', '', colnames(orthogroups), fixed=TRUE)
head(orthogroups)
#Add rownames
rownames(orthogroups)<-orthogroups$Orthogroup
orthogroups<-orthogroups[,-1]
head(orthogroups)
#Remove Total column
orthogroups$Total<-NULL
colnames(orthogroups)
```

2. Transform imput

However, this table is somewhat complex, and we only need a table with 0 (abscence) and 1 (presence) for each strain (in case some of we've missed to convert some number >1 to 1 in the preprocessing).

```
orthogroups[orthogroups > 1] <- 1
namerows<-rownames(orthogroups)
head(orthogroups)
orthogroups <- orthogroups %>% mutate_all(as.integer)
rownames(orthogroups)<-namerows
head(orthogroups)
```

As we can see in the example above, now all values in the table are either 0 or 1.

3. Index of random sampling

Now, we need to set a random indext of strain numbers in order to make replicates. In this case, we are going to use 300 replicates. This table will be used for all the calculations: core, strain-specific and pan-genome genomic fractions. We first need a text file with the name of each strain in a row, single column and the header “synonym”.

```
strains = read.table("strains_list.txt", header=T, sep="\t", colClasses = "chara
cter") #In case you don’t have this table, do not run this line and read the fol
lowing commands and uncomment the code.

#This table is just the numbers in which the 237 genomes are codified. It can
also be generated by running the following commands:

#strains = as.data.frame(c("1", "2", "4", "5", "6", "7", "9", "12", "13", "14",
"15", "17", "18", "19", "20", "21", "22", "23", "24", "25", "26", "27", "28", "2
9", "30", "31", "32", "33", "34", "36", "37", "38", "39", "40", "41", "42", "43"
, "45", "46", "47", "48", "49", "50", "51", "52", "53", "54", "55", "56", "57",
"58", "59", "63", "64", "65", "66", "67", "68", "69", "71", "78", "79", "81", "8
2", "85", "86", "87", "89", "90", "91", "96", "97", "98", "100", "101", "103", "
104", "105", "109", "110", "111", "115", "118", "119", "125", "126", "128", "129
", "130", "131", "132", "137", "138", "139", "140", "144", "145", "146", "149",
"150", "152", "153", "160", "168", "173", "175", "177", "179", "183", "184", "18
5", "186", "188", "189", "190", "191", "192", "193", "194", "195", "196", "199",
"201", "202", "204", "205", "206", "207", "208", "209", "210", "211", "213", "21
4", "215", "216", "217", "218", "219", "220", "221", "222", "223", "224", "225",
"226", "227", "229", "230", "231", "232", "233", "234", "235", "236", "237", "23
8", "239", "240", "241", "242", "244", "245", "246", "247", "249", "250", "252",
"253", "254", "255", "256", "257", "258", "259", "260", "262", "263", "264", "26
5", "266", "267", "268", "269", "270", "271", "272", "273", "274", "275", "276",
"277", "278", "279", "280", "281", "282", "283", "284", "285", "286", "288", "28
9", "290", "291", "292", "293", "294", "295", "296", "297", "298", "299", "300",
"301", "302", "306", "307", "308", "309", "310", "311", "313", "314", "315", "31
6", "317", "318", "319", "320", "321", "322", "323", "324", "325", "326", "327")
)
#colnames(strains) = "synonym"

strainsv = as.vector(strains$synonym)
strainsdf = as.data.frame(strainsv)

for (i in 1:300) { #300 Replicates
  strainsdf = cbind(strainsdf, sample(strainsv))
  tag = paste("model", i, sep="_")
  colnames(strainsdf)[i+1] <- tag
}
```

Let’s take a look into the sampling we’ve just created:

```
head(strainsdf)
```

Looks good, we have multiple rows, each one with a different model (from 1 to 300) and each model a randomly picked strain name from 1 to 237. We can continue now.

4. Genome fractions

We are going to randomly sample all genomes and ask about how many OGs are present according to core genome, specific-genome and pangenome. The code will perform all the calculations at once (looping throughout the straindf), which might take some time. Please, be pattient.

```
outtable_core<-data.frame(a=1:length(rownames(strainsdf)))
outtable_core$a<- NULL

outtable_pan<-data.frame(a=1:length(rownames(strainsdf)))
outtable_pan$a<- NULL

outtable_strain<-data.frame(a=1:length(rownames(strainsdf)))
outtable_strain$a<- NULL

for (i in 1:length(strainsdf)) {
  #print(paste("Loop", as.character(i), "of ", as.character(length(strainsdf))))
  coretable <- data.frame(a=1:length(rownames(orthogroups)))
  rownames(coretable) <- rownames(orthogroups)
  coretable$a<-NULL
  coreevol <- vector()
  panevol <- vector()
  strainspecevol <- vector()
  modelog <- as.vector(strainsdf[,i])
  for (strain in modelog) {
   coretable<- bind_cols(coretable, select(orthogroups, strain))
   core<-nrow(filter(coretable, rowSums(coretable)==length(colnames(coretable)))
)
   coreevol <- c(coreevol, core)
   pan<-nrow(filter(coretable, rowSums(coretable)>0))
   panevol <- c(panevol, pan)
   strainspec<-nrow(coretable %>% filter((!!as.symbol(strain)) == 1 & rowSums(co
retable)==1))
   strainspecevol <- c(strainspecevol, strainspec)
  }
  outtable_core <- cbind(outtable_core,coreevol)
  outtable_pan <- cbind(outtable_pan,panevol)
  outtable_strain <- cbind(outtable_strain,strainspecevol)
}
colnames(outtable_core)<-colnames(strainsdf)
colnames(outtable_pan)<-colnames(strainsdf)
colnames(outtable_strain)<-colnames(strainsdf)

#Save the tables in case we need to repeat something, to avoid making all the lo
ops again.
write.table(outtable_core, "CORE_EVOL.txt", quote = F, sep = "\t")
write.table(outtable_pan, "PAN_EVOL.txt", quote = F, sep = "\t")
write.table(outtable_strain, "STRAIN_EVOL.txt", quote = F, sep = "\t")
```

STATISTICS

We will compute now the mean, Q1 and Q3 statistics on the three tables before plotting them.

```
library("matrixStats")

core_mean = data.frame(Mean=rowMeans(outtable_core))
core_q1 = data.frame(Q1=rowQuantiles(as.matrix(outtable_core), probs = 0.25))
core_q3 = data.frame(Q3=rowQuantiles(as.matrix(outtable_core), probs = 0.75))
core_stats = cbind(Interation=c(1:237), core_mean, core_q1, core_q3)

pan_mean = data.frame(Mean=rowMeans(outtable_pan))
pan_q1 = data.frame(Q1=rowQuantiles(as.matrix(outtable_pan), probs = 0.25))
pan_q3 = data.frame(Q3=rowQuantiles(as.matrix(outtable_pan), probs = 0.75))
pan_stats = cbind(Interation=c(1:237), pan_mean, pan_q1, pan_q3)

strain_mean = data.frame(Mean=rowMeans(outtable_strain))
strain_q1 = data.frame(Q1=rowQuantiles(as.matrix(outtable_strain), probs = 0.25)
)
strain_q3 = data.frame(Q3=rowQuantiles(as.matrix(outtable_strain), probs = 0.75)
)
strain_stats = cbind(Interation=c(1:237), strain_mean, strain_q1, strain_q3)
```

CORE GENOME PLOT

```
library("ggplot2")
library(gridExtra)

c1 = ggplot(core_stats, aes(1:237, y = core_stats$Mean, ymin=core_stats$Q1, ymax
=core_stats$Q3)) +
  geom_line(color="seagreen", size=1) + ggtitle("Core (hard)") +
  geom_ribbon(alpha=0.2, fill="seagreen") + coord_cartesian(ylim = c(3500, 5000)
) +
  xlab("") + ylab("") + theme(axis.text.x=element_blank(), axis.ticks.x=element_
blank()) +
  theme(plot.margin=unit(c(0,0,-0.25,0), "cm"), axis.line.y=element_line())

c2 = ggplot(core_stats, aes(1:237, y = core_stats$Mean, ymin=core_stats$Q1, ymax
=core_stats$Q3)) +
  geom_line(color="seagreen", size=1) +
  geom_ribbon(alpha=0.2, fill="seagreen") + coord_cartesian(ylim = c(250, 3000))
+
  xlab("") + ylab("") + scale_y_continuous(breaks=c(250,500,750,1000,2000,2500,3
000)) +
  geom_hline(yintercept=1253, color="tomato3", linetype="dashed", size=0.8) + an
notate(geom="text", x=216, y=1323, label="99.16% (1253)", size=3.5) +
  geom_hline(yintercept=875, color="tomato3", linetype="dashed", size=0.65) + an
notate(geom="text", x=216, y=945, label="99.58% (875)", size=3.5) +
  geom_hline(yintercept=1493, color="tomato3", linetype="dashed", size=0.65) + a
nnotate(geom="text", x=216, y=1563, label="98.73% (1493)", size=3.5) +
  geom_point(x=237, y=381, size=4, colour="tomato3", pch = 1) + annotate(geom="t
ext", x=216, y=280, label="100% (381)", size=3.5) +
  theme(plot.margin=unit(c(0,0,0,0), "cm"), axis.line.x=element_line(), axis.lin
e.y=element_line())

grid_c = grid.arrange(c1, c2, heights = c(1.5,3))
```

PANGENOME PLOT

```
library("ggplot2")

pang = ggplot(pan_stats, aes(1:237, y = pan_stats$Mean, ymin=pan_stats$Q1, ymax=
pan_stats$Q3)) +
  geom_line(color="firebrick", size=1) + ggtitle("Pangenome") +
  geom_ribbon(alpha=0.2, fill="firebrick") + coord_cartesian(ylim = c(3000, 3000
0)) +
  xlab("") + ylab("") + scale_y_continuous(breaks=c(2500,5000,10000,15000,20000,
25000,30000)) +
  geom_point(x=237, y=26080, size=4, colour="firebrick", pch = 1) + annotate(geo
m="text", x=237, y=25200, label="20680", size=3.5) +
  theme(plot.margin=unit(c(0,0,0,0), "cm"), axis.line.x=element_line(), axis.lin
e.y=element_line())

pang
```

Strain-specific PLOT

```
s1 = ggplot(strain_stats, aes(1:237, y = strain_stats$Mean, ymin=strain_stats$Q1
, ymax=strain_stats$Q3)) +
  geom_line(color="dodgerblue4", size=1) + ggtitle("Strain-specific") +
  geom_ribbon(alpha=0.2, fill="dodgerblue4") + coord_cartesian(ylim = c(3000, 50
00)) +
  xlab("") + ylab("") + theme(axis.text.x=element_blank(), axis.ticks.x=element_
blank()) +
  theme(plot.margin=unit(c(0,0,-0.25,0), "cm"), axis.line.y=element_line())

s2 = ggplot(strain_stats, aes(1:237, y = strain_stats$Mean, ymin=strain_stats$Q1
, ymax=strain_stats$Q3)) +
  geom_line(color="dodgerblue4", size=1) +
  geom_ribbon(alpha=0.2, fill="dodgerblue4") + coord_cartesian(ylim = c(0, 1000)
) +
  xlab("") + ylab("") +
  theme(plot.margin=unit(c(0,0,0,0), "cm"), axis.line.x=element_line(), axis.lin
e.y=element_line())

grid_s = grid.arrange(s1, s2, heights = c(1.5,3))
```

PLOT ALL

```
grid.arrange(grid_s, grid_c, pang, top = "Genome fractions", left ="orthologous
sequences", bottom="Sampled genomes", ncol=3)
```
