# Supplementary material for: Comparative Genomics of the Rhodococcus Genus Shows Wide Distribution of Biodegradation Traits
Source: Microorganisms. 2020 May 21;8(5):774. doi: 10.3390/microorganisms8050774 (PMC7285261; doi:10.3390/microorganisms8050774)
Supplement: Supplementary file 1 [file microorganisms-08-00774-s001.zip › Supplementary_Table_S1.pdf]

Supplementary Table S1. List of *Rhodococcus* genomes used in this study.

Name	Type strain?	Assembly	Size (Mb)	GC%	Scaffolds	Genes	CDSs
R. aetherivorans BCP1	no	GCA_000470885.1	6.23	70.3	3	5,713	5,495
R. aetherivorans IcdP1	no	GCA_000982715.1	5.92	70.6	1	5,388	5,020
R. agglutinans CCTCC AB2014297	YES	GCA_004011865.1	5.43	69.2	22	4,988	4,835
R. baikonurensis JCM 18801	no	GCA_001311605.1	6.82	62.4	633	-	-
R. biphenylivorans TG9	YES	GCA_003288095.1	5.03	68	1	4,700	4,373
R. coprophilus NCTC 10994	YES	GCA_900478115.1	4.58	66.8	1	4,222	4,081
R. corynebacterioides NBRC 14404	YES	GCA_001894765.1	3.98	70.3	14	3655	3564
R. defluvii Ca11 T	YES	GCA_000738775.1	5.13	68.7	267	4,693	4,535
R. enclensis 23b-28	no	GCA_002744595.1	7.21	62.3	117	6828	6532
R. erythropolis 1159	no	GCA_002091935.1	7.09	62.3	112	6629	6359
R. erythropolis ACN1	no	GCA_002303875.1	7.24	62.3	92	6711	6479
R. erythropolis ATCC 15903	no	GCA_003388635.1	7.24	62.4	86	6870	6512
R. erythropolis AV96	no	GCA_002233715.1	6.44	62.4	45	6047	4918
R. erythropolis B7g	no	GCA_003444715.1	7.14	62.4	93	6696	6475
R. erythropolis BG43	no	GCA_000975175.1	6.87	62.3	4	6396	6233
R. erythropolis CAS922i	no	GCA_001020225.1	7.20	62.3	108	6727	6496
R. erythropolis CCM2595	no	GCA_000454045.1	6.37	62.5	2	5895	5776
R. erythropolis DN1	no	GCA_000454425.1	6.55	62.4	78	6152	5629
R. erythropolis IEGM 267	no	GCA_001900745.1	7.18	62.3	231	6745	6481
R. erythropolis JCM 3201	YES	GCA_003990875.1	6.65	62.4	3	6150	5976
R. erythropolis JCM 6824	no	GCA_000747745.1	7.02	62.3	198	6608	6372
R. erythropolis JCM 9803	no	GCA_001312725.1	6.87	62.3	212	-	-
R. erythropolis JCM 9804	no	GCA_001313245.1	6.55	62.4	260	6309	3200
R. erythropolis JCM 9805	no	GCA_001312745.1	6.96	62.4	235	6669	4261
R. erythropolis MI2	no	GCA_001766885.1	7.18	62.3	123	6765	6533
R. erythropolis NCTC8036	no	GCA_900455855.1	6.55	62.4	3	6044	5886
R. erythropolis NRRL B-16532	no	GCA_000719985.1	6.94	62.4	160	6522	6300
R. erythropolis NSX2	no	GCA_001715845.1	6.28	62.4	37	5820	5692
R. erythropolis PR4	no	GCA_000010105.1	6.90	62.3	4	6491	6321
R. erythropolis R138	no	GCA_000696675.2	6.81	62.3	3	6301	6130
R. erythropolis S-43	no	GCA_000830355.1	6.81	62.2	533	6678	3687
R. erythropolis SK121	no	GCA_000174835.1	6.79	62.5	124	6369	6158
R. erythropolis VSD3	no	GCA_001831305.1	6.55	62.4	38	6064	5659
R. erythropolis XP	no	GCA_000225665.2	7.23	62.3	9	6826	6569
R. fascians 02-815	no	GCA_000760835.1	6.24	64.4	30	5817	5674
R. fascians 02-816c	no	GCA_000760855.1	6.08	64.6	45	5830	5674
R. fascians 04-516	no	GCA_000760685.1	5.82	64.3	23	5472	5315
R. fascians 05-339-1	no	GCA_000760895.1	5.73	64.7	21	5454	5307
R. fascians 05-561-1	no	GCA_000760875.1	5.61	64.5	30	5311	5187
R. fascians 14-2632c-1	no	GCA_002259325.1	5.95	64.4	41	5639	5477
R. fascians 14-2632-D2	no	GCA_002259295.1	5.93	64.4	44	5621	5459
R. fascians 15-508-1b	no	GCA_002258705.1	5.95	64.4	41	5637	5478
R. fascians A2	no	GCA_002259505.1	5.97	64.4	44	5662	5500
R. fascians A21d2	no	GCA_000760905.1	5.98	64.1	30	5624	5470
R. fascians A22b	no	GCA_000759005.1	5.91	64.3	67	5574	5354
R. fascians A25f	no	GCA_000760935.1	5.87	64.2	17	5551	5404
R. fascians A3b	no	GCA_000760675.1	6.03	64.3	34	5758	5579
R. fascians A44A	no	GCA_000760735.1	5.95	64.6	9	5564	5416
R. fascians A73a	no	GCA_000760755.1	5.93	64.4	23	5489	5333
R. fascians A76	no	GCA_000760955.1	6.03	64.6	29	5709	5566
R. fascians A78	no	GCA_000760775.1	6.00	64.4	41	5659	5503
R. fascians B3	no	GCA_002259465.1	5.93	64.4	46	5621	5461
R. fascians D188	no	GCA_001620305.1	5.50	64.6	3	5149	5015
R. fascians F7	no	GCA_001037935.1	5.25	64.7	21	4929	4819
R. fascians GIC26	no	GCA_000760795.1	5.33	64.5	49	4991	4831
R. fascians GIC36	no	GCA_000760815.1	5.56	64.5	46	5232	5060
R. fascians LMG 3602	no	GCA_000760995.1	5.36	64.6	25	5113	4961
R. fascians LMG 3605	no	GCA_000761015.1	5.44	64.6	28	5123	4976
R. fascians LMG 3616	no	GCA_000761035.1	5.76	64.4	42	5455	5283
R. fascians LMG 3623	YES	GCA_000761055.1	5.77	64.4	30	5427	5271
R. fascians LMG 3625	no	GCA_000761075.1	5.94	64.2	17	5719	5576
R. globerulus NBRC 14531	YES	GCA_001894805.1	6.74	61.7	30	6231	6054
R. globerulus WS3306	no	GCA_003097035.1	6.77	61.7	2	6220	5996
R. gordoniae NCTC13296	YES	GCA_900455725.1	4.87	67.9	3	4,458	4,287
R. hoagii 103S	no	GCA_000196695.1	5.04	68.8	1	4,649	4,540
R. hoagii ATCC 33707	no	GCA_000164155.2	5.26	68.7	1	4,899	4,775
R. hoagii DSM 20295	YES	GCA_001646645.1	4.97	68.8	279	4,757	4,427

R. hoagii DSM 20307	no	GCA_002094305.1	5.20	68.8	37	4,850	4,735
R. hoagii DSSKP-R-001	no	GCA_003013675.1	5.44	68.7	3	5,118	4,987
R. hoagii N1288	no	GCA_001646885.1	5.17	68.8	47	4,845	4,685
R. hoagii N1295	no	GCA_001646905.1	5.31	68.7	156	5,034	4,769
R. hoagii N1301	no	GCA_001646925.1	5.65	68.5	61	5,297	5,099
R. hoagii NBRC 101255 - C 7	no	GCA_001552575.1	5.20	68.8	48	4,879	4,725
R. hoagii NCTC1621	no	GCA_900455845.1	5.24	68.8	3	4,885	4,760
R. hoagii NCTC5650	no	GCA_900455885.1	5.31	68.7	7	4,962	4,785
R. hoagii PAM1204	no	GCA_002078545.1	5.23	68.8	42	4,829	4,713
R. hoagii PAM1216	no	GCA_002095175.1	5.20	68.8	41	4,838	4,719
R. hoagii PAM1271	no	GCA_002095045.1	5.20	68.8	41	4,849	4,732
R. hoagii PAM1340	no	GCA_002095085.1	5.06	68.8	25	4,710	4,599
R. hoagii PAM1354	no	GCA_002095035.1	5.37	68.7	37	4,983	4,862
R. hoagii PAM1357	no	GCA_002095125.1	5.01	68.8	24	4,658	4,547
R. hoagii PAM1413	no	GCA_002095115.1	5.04	68.8	39	4,693	4,558
R. hoagii PAM1422	no	GCA_002095195.1	5.20	68.8	41	4,842	4,729
R. hoagii PAM1475	no	GCA_002095185.1	5.21	68.8	60	4,823	4,703
R. hoagii PAM1496	no	GCA_002095155.1	5.09	68.8	41	4,726	4,603
R. hoagii PAM1533	no	GCA_002095235.1	5.13	68.8	82	4,814	4,657
R. hoagii PAM1557	no	GCA_002095255.1	5.35	68.7	34	4,976	4,851
R. hoagii PAM1571	no	GCA_002094265.1	5.42	68.6	61	5,027	4,886
R. hoagii PAM1572	no	GCA_002078535.1	5.12	68.8	29	4,735	4,631
R. hoagii PAM1593	no	GCA_002078625.1	5.24	68.7	49	4,865	4,739
R. hoagii PAM1600	no	GCA_002078515.1	5.14	68.8	41	4,757	4,661
R. hoagii PAM1637	no	GCA_002095295.1	5.08	68.9	45	4,686	4,588
R. hoagii PAM1643	no	GCA_002094235.1	5.09	68.8	31	4,765	4,650
R. hoagii PAM2012	no	GCA_002094445.1	5.33	68.7	31	4,932	4,815
R. hoagii PAM2274	no	GCA_002094225.1	5.22	68.7	44	4,869	4,745
R. hoagii PAM2276	no	GCA_002094315.1	5.61	68.6	840	5,192	4,879
R. hoagii PAM2279	no	GCA_002094375.1	5.03	68.8	32	4,693	4,578
R. hoagii PAM2282	no	GCA_002094295.1	5.27	68.7	41	4,887	4,791
R. hoagii PAM2285	no	GCA_002094395.1	5.19	68.8	40	4,814	4,708
R. hoagii PAM2287	no	GCA_002094405.1	5.14	68.9	40	4,744	4,643
R. hoagii PAM2288	no	GCA_002094325.1	5.48	68.7	182	5,127	4,967
R. imtechensis RKJ300	YES	GCA_000260815.1	8.23	67.2	178	7,962	7,245
R. jostii DSM 44719	no	GCA_900105375.1	9.91	66.9	6	9,052	8,473
R. jostii NBRC 16295	YES	GCA_001894825.1	9.73	66.9	286	8,983	8,358
R. jostii RHA1	no	GCA_000014565.1	9.70	67	4	9,256	8,690
R. koreensis DSM 44498	YES	GCA_900105905.1	10.31	67.4	9	9,491	8,902
R. kroppenstedtii DSM 44908	YES	GCA_900111805.1	4.08	70.1	30	3789	3652
R. kunmingensis DSM 45001	YES	GCA_001646865.1	5.62	66.2	330	5,268	4,885
R. kyotonensis JCM 23211	YES	GCA_900188125.1	6.31	64.2	50	5860	5698
R. kyotonensis KB10	no	GCA_001645385.1	5.47	65.2	41	5056	4893
R. maanshanensis DSM 44675	YES	GCA_900109405.1	5.67	69.2	61	5,143	4,972
R. marinonascens NBRC 14363	YES	GCA_001894885.1	4.92	64.4	156	4619	4269
R. opacus 04-OD7	no	GCA_002968035.1	9.32	66.9	220	8,884	8,099
R. opacus 1CP	no	GCA_001685605.1	8.64	67	3	7,973	7,380
R. opacus 8	no	GCA_001292845.1	8.51	67.2	507	8,310	7,401
R. opacus ATCC 51882	YES	GCA_004365075.1	9.87	66.8	3	9,215	8,486
R. opacus B4	no	GCA_000010805.1	8.83	67.6	6	8,227	7,837
R. opacus DSM 44186	no	GCA_003626495.1	8.81	67.1	2	8,261	7,611
R. opacus M213	no	GCA_000264745.2	9.19	67	483	8,743	7,979
R. opacus NRRL B-24011	no	GCA_000719995.1	6.38	62.4	334	5835	5672
R. opacus PD630	no	GCA_000599545.1	9.17	67.2	10	8,402	7,903
R. opacus R7	no	GCA_000736435.1	10.12	66.9	6	9,273	8,731
R. phenolicus DSM 44812	YES	GCA_001646785.1	6.28	68.4	232	5,916	5,600
R. pyridinivorans AK37	no	GCA_000236965.2	5.24	67.9	98	4,938	4,482
R. pyridinivorans DSM 44555	YES	GCA_900105195.1	5.26	67.8	3	4,864	4,604
R. pyridinivorans GF3	no	GCA_002269365.1	5.30	67.9	1	4,966	4,644
R. pyridinivorans KG-16	no	GCA_001465325.1	5.83	67.7	87	5,286	5,074
R. pyridinivorans SB3094	no	GCA_000511305.1	5.59	67.8	3	5,165	4,893
R. pyridinivorans YF3	no	GCA_005944105.1	6.09	-	5	-	-
R. pyridinivorans ZKA33	no	GCA_003633655.1	5.71	67.7	4	5,371	5,011
R. pyridinivorans ZKA49	no	GCA_003610315.1	6.42	67.6	4	5,850	5,626
R. qingshengii BKS 20-40	no	GCA_000341815.1	6.60	62.4	104	6208	5999
R. qingshengii CS98	no	GCA_001662505.1	6.71	62.4	25	6278	6096
R. qingshengii CW25	no	GCA_001623435.1	6.40	62.5	10	6022	5876
R. qingshengii JCM 15477	YES	GCA_001646745.1	7.26	62.4	131	6820	6574
R. qingshengii MK1	no	GCA_002087025.1	6.47	62.5	40	6039	5871
R. qingshengii TUHH-12	no	GCA_000698455.1	7.43	61.7	349	7064	5249

R. rhodnii NBRC 100604	YES	GCA_001894925.1	4.46	69.7	70	4,265	4,080
R. rhodochrous 11Y	no	GCA_002003765.1	8.58	70.4	1809	8,676	7,062
R. rhodochrous ATCC 17895	no	GCA_000469645.1	6.87	62.3	423	6396	6122
R. rhodochrous ATCC 21198	no	GCA_000517665.1	6.48	70.2	161	5,957	5,692
R. rhodochrous BKS6-46	no	GCA_000239135.3	6.21	67.4	609	5,664	5,348
R. rhodochrous EP4	no	GCA_003004765.2	5.72	67.9	1	5,198	4,942
R. rhodochrous J3	no	GCA_900177695.1	6.11	67.9	68	5,576	5,343
R. rhodochrous KG-21	no	GCA_001278665.1	6.10	69.6	232	5,633	5,215
R. rhodochrous NCTC 10210	YES	GCA_900187265.1	5.27	68.2	1	4,841	4,668
R. rhodochrous NCTC 630	no	GCA_900455745.1	6.51	70.6	2	6,049	5,808
R. rhodochrous NRRL B-1306	no	GCA_000716895.1	6.78	61.7	85	6293	6074
R. rhodochrous TRN71	no	GCA_001511235.1	4.87	70.2	173	4,594	3,147
R. ruber BKS 20-38	no	GCA_000341965.1	6.13	69.7	108	5,642	5,342
R. ruber Chol-4	no	GCA_000347955.2	5.46	70.7	44	5,001	4,861
R. ruber IEGM 231	no	GCA_000824945.2	6.00	70.2	46	5,551	5,294
R. ruber NBRC 15591	YES	GCA_001894945.1	5.33	70.7	56	4,879	4,740
R. ruber OA1	no	GCA_003844175.1	5.58	70.3	323	5,266	4,878
R. ruber P14	no	GCA_002741725.1	5.52	70.5	1	5,053	4,869
R. ruber P25	no	GCA_001051275.1	5.73	70.5	65	5,248	5,088
R. ruber SD3	no	GCA_003086595.1	5.37	70.6	1	4,905	4,768
R. ruber YC-YT1	no	GCA_003586525.1	5.91	70.2	3	5,392	5,181
R. ruber YYL	no	GCA_002863905.1	5.92	70.3	3	5,497	5,266
Rhodococcus sp. 008	no	GCA_001682295.1	7.44	62.3	4	6964	6767
Rhodococcus sp. 02-925g	no	GCA_002259105.1	5.75	64.7	38	5394	5245
Rhodococcus sp. 05-2221-1B	no	GCA_002258635.1	6.38	64.8	60	6056	5880
Rhodococcus sp. 05-2254-1	no	GCA_002258625.1	5.93	64.5	35	5620	5497
Rhodococcus sp. 05-2254-2	no	GCA_002259095.1	5.89	64.6	25	5575	5431
Rhodococcus sp. 05-2254-3	no	GCA_002258555.1	5.89	64.6	33	5585	5432
Rhodococcus sp. 05-2254-4	no	GCA_002259035.1	5.92	64.6	30	5615	5465
Rhodococcus sp. 05-2254-5	no	GCA_002258575.1	5.74	64.5	35	5425	5303
Rhodococcus sp. 05-2254-6	no	GCA_002258565.1	5.66	64.7	195	5485	5107
Rhodococcus sp. 05-2255-1e	no	GCA_002259025.1	5.75	64.5	51	5432	5277
Rhodococcus sp. 05-2255-2A2	no	GCA_002258995.1	5.70	64.7	56	5432	5264
Rhodococcus sp. 05-2255-3B1	no	GCA_002258545.1	5.73	64.7	46	5474	5314
Rhodococcus sp. 05-2255-3C	no	GCA_002258515.1	5.73	64.7	63	5469	5296
Rhodococcus sp. 05-2256-B1	no	GCA_002258465.1	5.77	64.4	42	5473	5313
Rhodococcus sp. 05-2256-B2	no	GCA_002258485.1	5.77	64.4	49	5477	5311
Rhodococcus sp. 05-2256-B3	no	GCA_002259065.1	5.77	64.4	42	5466	5313
Rhodococcus sp. 05-2256-B4	no	GCA_002258975.1	5.77	64.4	62	5485	5316
Rhodococcus sp. 05-339-2	no	GCA_002258965.1	5.73	64.8	22	5446	5299
Rhodococcus sp. 05-340-1	no	GCA_002258925.1	5.98	64.6	44	5661	5512
Rhodococcus sp. 05-340-2	no	GCA_002258915.1	5.98	64.6	43	5655	5510
Rhodococcus sp. 06-1059B-a	no	GCA_002258905.1	5.68	64.7	73	5305	5121
Rhodococcus sp. 06-1460-1B	no	GCA_002258475.1	5.78	64.6	46	5496	5367
Rhodococcus sp. 06-1474-1B	no	GCA_002258885.1	5.34	64.6	37	5009	4861
Rhodococcus sp. 06-1477-1A	no	GCA_002258865.1	6.05	64.6	33	5681	5520
Rhodococcus sp. 06-1477-1B	no	GCA_002258825.1	8.84	66.7	115	8296	7998
Rhodococcus sp. 06-156-3	no	GCA_002258835.1	6.30	64.4	42	5857	5727
Rhodococcus sp. 06-156-3b	no	GCA_002258445.1	6.31	64.3	48	5867	5737
Rhodococcus sp. 06-156-3C	no	GCA_002258795.1	6.36	64.3	46	5941	5808
Rhodococcus sp. 06-156-4	no	GCA_002259345.1	6.41	64.4	39	5972	5840
Rhodococcus sp. 06-156-4a	no	GCA_002258405.1	6.30	64.4	45	5856	5723
Rhodococcus sp. 06-156-4C	no	GCA_002258395.1	6.30	64.4	47	5857	5723
Rhodococcus sp. 06-221-2	no	GCA_002258365.1	5.68	64.5	38	5294	5181
Rhodococcus sp. 06-235-1A	no	GCA_002258785.1	6.10	64.3	37	5810	5644
Rhodococcus sp. 06-412-2B	no	GCA_002258385.1	6.36	64	30	6005	5859
Rhodococcus sp. 06-412-2C	no	GCA_002258295.1	6.21	64	30	5860	5712
Rhodococcus sp. 06-418-1B	no	GCA_002258765.1	6.13	64.6	55	5734	5577
Rhodococcus sp. 06-418-5	no	GCA_002258285.1	6.06	64.7	39	5749	5597
Rhodococcus sp. 06-462-5	no	GCA_002258315.1	5.76	64.7	61	5408	5241
Rhodococcus sp. 06-469-3-2	no	GCA_002258305.1	6.03	64.6	36	5664	5496
Rhodococcus sp. 06-470-2	no	GCA_002258235.1	5.90	64.8	50	5605	5442
Rhodococcus sp. 06-621-2	no	GCA_002258205.1	6.23	64.5	67	5881	5703
Rhodococcus sp. 1139	no	GCA_001757665.1	7.04	62.3	187	6700	6396
Rhodococcus sp. 114MFTsu3.1	no	GCA_000383555.1	5.55	64.7	35	5177	5052
Rhodococcus sp. 1163	no	GCA_002091985.1	4.46	62.3	43	4099	3953
Rhodococcus sp. 1168	no	GCA_002091955.1	5.06	62.1	97	4663	4421
Rhodococcus sp. 14-1411-2a	no	GCA_002259485.1	5.61	64.7	27	5288	5147
Rhodococcus sp. 14-2470-1a	no	GCA_002259425.1	6.07	64.6	67	5680	5494
Rhodococcus sp. 14-2470-1b	no	GCA_002259415.1	5.91	64.4	73	5533	5341

Rhodococcus sp. 14-2483-1-1	no	GCA_002259335.1	5.78	64.3	36	5421	5292
Rhodococcus sp. 14-2483-1-2	no	GCA_002259405.1	5.87	64.6	27	5464	5295
Rhodococcus sp. 14-2496-1d	no	GCA_002259365.1	5.92	64.8	55	5683	5510
Rhodococcus sp. 14-2686-1-2	no	GCA_002259285.1	6.20	64.3	58	5855	5646
Rhodococcus sp. 15-1154-1	no	GCA_002258715.1	5.98	64.5	47	5614	5438
Rhodococcus sp. 15-1189-1-1a	no	GCA_002259155.1	6.24	64.3	69	5894	5679
Rhodococcus sp. 15-2388-1-1a	no	GCA_002259145.1	6.21	64.5	55	5896	5732
Rhodococcus sp. 15-649-1-2	no	GCA_002258685.1	5.78	64.6	42	5393	5269
Rhodococcus sp. 15-649-2-2	no	GCA_002258645.1	6.03	64.1	52	5726	5587
Rhodococcus sp. 15-725-2-2b	no	GCA_002259075.1	6.12	64.6	33	5738	5577
Rhodococcus sp. 164Chir2E	no	GCA_900101565.1	6.26	62.5	46	5884	5718
Rhodococcus sp. 1R11	no	GCA_004563845.1	5.48	64.5	38	5156	4989
Rhodococcus sp. 29MFTsu3.1	no	GCA_000382105.1	5.58	64.6	57	5250	5102
Rhodococcus sp. 2G	no	GCA_001886355.1	5.60	67.5	2	5,256	4,930
Rhodococcus sp. 311R	no	GCA_001242945.1	6.34	62.6	128	5935	5735
Rhodococcus sp. 4J2A2	no	GCA_001373455.1	6.44	61.8	60	5988	5762
Rhodococcus sp. 66b	no	GCA_002076325.1	6.68	62.4	57	6253	6069
Rhodococcus sp. 852002-51564 SCH6189132-a	no	GCA_001665495.1	4.86	68.2	87	4,504	4,250
Rhodococcus sp. ABRD24	no	GCA_004328705.1	5.10	66.4	1	4,572	4,381
Rhodococcus sp. ACPA1	no	GCA_002300195.1	10.06	66.9	47	9,292	8,798
Rhodococcus sp. ACPA4	no	GCA_002300185.1	7.07	61.6	9	6475	6269
Rhodococcus sp. ACS1	no	GCA_002300155.1	10.89	67	40	10,089	9,459
Rhodococcus sp. AD45	no	GCA_000949305.1	6.79	61.7	9	6252	6070
Rhodococcus sp. AD45-ID	no	GCA_003023755.1	6.45	61.8	8	5957	5787
Rhodococcus sp. ADH	no	GCA_001297885.1	7.08	62.3	129	6602	6389
Rhodococcus sp. AG1013	no	GCA_003350365.1	5.86	68.2	55	5,585	5,258
Rhodococcus sp. AJR001	no	GCA_001652355.1	7.26	62.4	167	6833	6556
Rhodococcus sp. AQ5-07	no	GCA_003313445.1	6.75	62.4	33	6352	6149
Rhodococcus sp. ARP2	no	GCA_001029585.1	6.30	62.4	120	5946	5730
Rhodococcus sp. AW25M09	no	GCA_000333955.1	5.64	64.1	167	5384	5101
Rhodococcus sp. B7740	no	GCA_000954115.1	5.34	64.9	1	5018	4875
Rhodococcus sp. BH4	no	GCA_002079265.1	7.02	62.3	2	6533	6362
Rhodococcus sp. Br-6	no	GCA_001748445.2	5.50	68.7	23	5,155	5,012
Rhodococcus sp. BS-15	no	GCA_000813105.1	5.50	64.4	656	5547	4101
Rhodococcus sp. BUPNP1	no	GCA_002215235.1	5.56	68.1	89	5,092	4,924
Rhodococcus sp. C9-28	no	GCA_005434945.1	4.40	69.1	67	-	-
Rhodococcus sp. Chr-9	no	GCA_000801125.1	5.34	67.7	142	5,047	4,651
Rhodococcus sp. CUA-806	no	GCA_001942265.1	5.80	63.9	67	5645	3966
Rhodococcus sp. D-1	no	GCA_001976025.1	6.93	62.3	57	6535	6356
Rhodococcus sp. DK17	no	GCA_000263875.1	9.11	67.1	135	8,611	7,954
Rhodococcus sp. ENV425	no	GCA_002887785.1	6.21	70.3	313	5,819	5,171
Rhodococcus sp. EPR-134	no	GCA_001647205.1	6.99	62.4	811	6928	6120
Rhodococcus sp. EPR-147	no	GCA_001647185.1	5.35	64.8	596	5363	4773
Rhodococcus sp. EPR-157	no	GCA_001647195.1	5.94	63.5	695	5840	5203
Rhodococcus sp. EPR-279	no	GCA_001647175.1	5.35	64.8	602	5366	4750
Rhodococcus sp. EsD8	no	GCA_000382865.1	6.63	70.1	287	6,081	5,747
Rhodococcus sp. Eu-32	no	GCA_003336925.1	5.61	65.1	83	5236	5079
Rhodococcus sp. HA99	no	GCA_001312925.1	5.89	65.4	1267	-	-
Rhodococcus sp. H-CA8f	no	GCA_002501585.1	6.50	62.5	2	6122	5979
Rhodococcus sp. HS-D2	no	GCA_001651055.1	5.75	67.5	202	5,432	5,103
Rhodococcus sp. IITR03	no	GCA_001017865.1	6.29	67.3	657	6,123	2,676
Rhodococcus sp. JCM 9791	no	GCA_001312645.1	6.34	65.3	947	-	-
Rhodococcus sp. JCM 9793	no	GCA_001312665.1	6.28	65.3	1101	-	-
Rhodococcus sp. JG-3	no	GCA_000482405.1	5.29	64.5	6	4968	4848
Rhodococcus sp. JVH1	no	GCA_000280725.1	9.18	67	173	8,610	8,073
Rhodococcus sp. KB6	no	GCA_001445685.1	7.00	62.5	165	6578	6348
Rhodococcus sp. KBS0724	no	GCA_005938745.1	7.85	59.5	1556	8118	7831
Rhodococcus sp. KBW08	no	GCA_003852455.1	6.95	62.4	87	6405	6206
Rhodococcus sp. LB1	no	GCA_001583455.1	10.75	66.6	448	10,008	9,213
Rhodococcus sp. Leaf225	no	GCA_001426145.1	4.71	68.3	26	4402	4274
Rhodococcus sp. Leaf233	no	GCA_001426165.1	5.53	64.5	15	5209	5089
Rhodococcus sp. Leaf247	no	GCA_001426185.1	4.53	67.1	5	4241	4119
Rhodococcus sp. Leaf258	no	GCA_001426065.1	4.71	68.4	19	4412	4293
Rhodococcus sp. Leaf278	no	GCA_001426085.1	5.72	64.1	45	5386	5215
Rhodococcus sp. Leaf7	no	GCA_001425985.1	4.54	67.1	7	4244	4120
Rhodococcus sp. LHW50502	no	GCA_004011835.1	3.97	66.5	29	3,607	3,453
Rhodococcus sp. LHW51113	no	GCA_004011825.1	3.71	66.5	22	3,373	3,243
Rhodococcus sp. LP 11 YM	no	GCA_004364595.1	7.42	62.2	63	6969	6671
Rhodococcus sp. LP 3 YM	no	GCA_004363395.1	7.42	62.2	65	6967	6671

Rhodococcus sp. M8	no	GCA_001890475.2	6.32	70.3	13	5,746	5,561
Rhodococcus sp. MEB064	no	GCA_000834565.1	4.66	67	81	4373	4239
Rhodococcus sp. MTM3W5.2	no	GCA_001984015.1	5.67	69	1	5,165	4,272
Rhodococcus sp. NCIMB 12038	no	GCA_002165735.1	9.27	67.2	109	8,498	8,035
Rhodococcus sp. NEAU-CX67	no	GCA_005049235.1	5.37	-	31	-	-
Rhodococcus sp. NJ-530	no	GCA_003860625.1	7.32	62.3	5	6899	6491
Rhodococcus sp. OK269	no	GCA_003386655.1	6.08	63.2	30	5706	5522
Rhodococcus sp. OK270	no	GCA_900215125.1	5.73	69	42	5,356	5,143
Rhodococcus sp. OK302	no	GCA_002245895.1	6.79	61.3	2	6315	5976
Rhodococcus sp. OK519	no	GCA_003051005.1	5.28	67.8	28	4,895	4,727
Rhodococcus sp. OK551	no	GCA_003387295.1	7.02	61.7	63	6570	6314
Rhodococcus sp. OK611	no	GCA_003050885.1	5.73	69	41	5,349	5,147
Rhodococcus sp. P1Y	no	GCA_003641205.1	5.87	63.2	1	5453	5253
Rhodococcus sp. P27	no	GCA_000454285.1	6.26	62.4	60	5956	4982
Rhodococcus sp. p52	no	GCA_000763325.2	5.41	67.8	4	5,076	4,753
Rhodococcus sp. PAMC28705	no	GCA_004795875.1	4.73	62.1	1	4302	4117
Rhodococcus sp. PAMC28707	no	GCA_004795915.1	4.73	62.1	1	4307	4122
Rhodococcus sp. PBTS 1	no	GCA_001620025.1	4.25	70	1	3918	3793
Rhodococcus sp. PBTS 2	no	GCA_001620005.1	5.32	64.7	2	4985	4864
Rhodococcus sp. PMG 084	no	GCA_004296005.1	2.78	66.8	407	2,931	2,620
Rhodococcus sp. PMG 254	no	GCA_004211705.1	6.90	61.7	63	6323	6103
Rhodococcus sp. PMG 259	no	GCA_004211695.1	8.47	66.6	1043	8,392	7,409
Rhodococcus sp. PML026	no	GCA_000963615.1	5.18	64.6	16	4840	4694
Rhodococcus sp. Q1	no	GCA_004153645.1	4.97	67.9	26	4,552	4,367
Rhodococcus sp. R04	no	GCA_000219395.2	8.84	69.6	2113	-	-
Rhodococcus sp. R1101	no	GCA_000278445.1	4.65	68	990	4,606	3,765
Rhodococcus sp. RD6.2	no	GCA_001040705.1	5.57	68.4	13	5,089	4,947
Rhodococcus sp. RS1C4	no	GCA_002258225.1	5.92	64.5	79	5558	5415
Rhodococcus sp. S2-17	no	GCA_003130705.1	8.01	65.6	4	7,531	6,877
Rhodococcus sp. SBT000017	no	GCA_003688915.1	5.65	64.6	5	5399	5169
Rhodococcus sp. SC4	no	GCA_001555475.1	10.57	66.7	345	9,793	9,118
Rhodococcus sp. UNC23MFCrub1.1	no	GCA_000686025.1	4.70	68.5	24	4394	4258
Rhodococcus sp. UNC363MFTsu5.1	no	GCA_000686785.1	5.67	69.3	69	5,257	5,079
Rhodococcus sp. WAY2	no	Unpublished	8.44	65.8	5	8,236	7,631
Rhodococcus sp. WB1	no	GCA_001700945.1	6.15	70.5	2	5,565	5,390
Rhodococcus sp. WMMA185	no	GCA_001767395.1	4.44	64.1	1	4014	3885
Rhodococcus sp. WWJCD1	no	GCA_002258735.1	5.86	64.2	41	5505	5352
Rhodococcus sp. X156	no	GCA_004006015.1	3.72	72.2	1	3504	3389
Rhodococcus sp. YH3-3	no	GCA_001653035.1	7.32	62.1	38	6967	6613
Rhodococcus sp. YL-0	no	GCA_002165495.1	7.23	62.4	174	6800	6548
Rhodococcus sp. YL-1	no	GCA_001942025.1	7.59	62.4	6	7109	6887
R. triatomae BKS 15-14	no	GCA_000341795.1	5.82	69	74	5,340	5,183
R. triatomae DSM 44892	YES	GCA_900099725.1	4.73	68.7	38	4,430	4,297
R. tukisamuensis JCM 11308	YES	GCA_900101735.1	5.49	69.8	40	4,976	4,759
R. wratislaviensis C31-06	no	GCA_003851765.1	9.54	67.2	193	8,914	8,364
R. wratislaviensis IFP 2016	no	GCA_000325625.1	9.69	67	927	9,777	8,187
R. wratislaviensis NBRC 100605	YES	GCA_000583735.1	10.40	66.8	151	9,514	8,883
R. wratislaviensis NCTC 13229	no	GCA_900455735.1	7.78	67.4	29	7,174	6,835
R. wratislaviensis WS3308	no	GCA_003385055.1	7.84	67.4	1	7,200	6,890
R. yunnanensis NBRC 103083	YES	GCA_001895005.1	6.37	63.9	68	5826	5650
R. zopfii NBRC 100606	YES	GCA_001895025.1	6.30	68.2	146	5,848	5,575
